# Protein Truncating Variants of *colA* in *Clostridium perfringens* Type G Strains

**DOI:** 10.3389/fcimb.2021.645248

**Published:** 2021-04-29

**Authors:** Lore Van Damme, Natasja Cox, Chana Callens, Michelle Dargatz, Monika Flügel, Sarah Hark, Frank Thiemann, Stefan Pelzer, Freddy Haesebrouck, Richard Ducatelle, Filip Van Immerseel, Evy Goossens

**Affiliations:** ^1^ Livestock Gut Health Team Ghent, Department of Pathology, Bacteriology and Avian Diseases, Faculty of Veterinary Medicine, Ghent University, Merelbeke, Belgium; ^2^ Evonik Operations GmbH, Division Nutrition & Care – Animal Nutrition, Westfalen, Germany

**Keywords:** *Clostridium perfringens*, kappa toxin, collagenase, nonsense mutation, necrotic enteritis disease

## Abstract

Extracellular matrix (ECM) degrading enzymes produced by *Clostridium perfringens* may play an important role during the initial phases of avian necrotic enteritis by facilitating toxin entry in the intestinal mucosa and destruction of the tissue. *C. perfringens* is known to produce several ECM-degrading proteases, such as kappa toxin, an extracellular collagenase that is encoded by the *colA *gene. In this study, the *colA* gene sequence of a collection of 48 *C. perfringens *strains, including pathogenic (i.e. toxinotype G) and commensal (i.e. toxinotype A) chicken derived strains and strains originating from other host species, was analyzed. Although the *colA* gene showed a high level of conservation (>96% nucleotide sequence identity), several gene variants carrying different nonsense mutations in the *colA* gene were identified, leading to the definition of four truncated collagenase variant types (I-IV). Collagenase variant types I, III and IV have a (nearly) complete collagenase unit but lack parts of the C-terminal recruitment domains, whereas collagenase variant types II misses the N-terminal part of collagenase unit. Gene fragments encoding a truncated collagenase were mainly linked with necrotic enteritis associated *C. perfringens* type G strains with collagenase variant types I and II being the most prevalent types. Gelatin zymography revealed that both recombinant full-length and variant type I collagenase have active auto-cleavage products. Moreover, both recombinant fragments were capable of degrading type I as well as type IV collagen, although variant type I collagenase showed a higher relative activity against collagen type IV as compared to full-length collagenase. Consequently, these smaller truncated collagenases might be able to break down collagen type IV in the epithelial basement membrane of the intestinal villi and so contribute to the initiation of the pathological process leading to necrotic enteritis.

## Introduction

Collagens are major glycoproteins of the extracellular matrix and play an important role in maintaining the biological and structural integrity of various tissues and organs in both humans and animals (Rozario and DeSimone, 2010). Being ubiquitously present, collagens are attractive targets for various microorganisms to induce tissue destruction (Bober et al., 2010; Singh et al., 2012). Indeed, many different bacterial pathogens, including members of the *Clostridium* genus, have been shown to produce collagenolytic enzymes (Harrington, 1996; Duarte et al., 2016). By digesting the collagen-rich extracellular matrix (ECM), pathogens can facilitate their own colonization and spread through the host tissues (Matsushita and Okabe, 2001; Duarte et al., 2016). Additionally, digestion of ECM compounds contributes to the availability of amino acids for the pathogen’s survival and growth and can also favor toxin diffusion and tissue damage (Kalisz, 1988; Harrington, 1996; Singh et al., 2012; Tomlin and Piccinini, 2018).

*Clostridium (C.) perfringens* is one of the collagenase-producing *Clostridia* and is known to cause a variety of histotoxic and enterotoxic diseases in both humans and animals (Niilo, 1980; Uzal et al., 2010). Although toxicity of *C. perfringens* is mostly dependent on the production of specific toxins, extracellular proteolytic enzymes are thought to assist in the development of some diseases. *C. perfringens* has been shown to produce multiple gelatinolytic enzymes with molecular masses ranging from ~80* *to approximately* *~120 kDa (Kameyama and Akama, 1971). The 80 kDa collagenase, which was formerly designated as kappa toxin, is lethal for mice upon intravenous injection and has both hemorrhagic and dermonecrotic activities upon subcutaneous injection in guinea pigs (Kameyama and Akama, 1971). Since the mid-90s, the focus of scientific research has shifted from the 80 to the 120 kDa collagenase and the term kappa toxin was revised, now denoting the ~120 kDa enzyme (Obana et al., 2010; Obana et al., 2013). This enzyme is encoded by the chromosomal *colA* gene and is synthesized as a precursor enzyme (pre-pro-collagenase; 126 kDa) with an N-terminal stretch of 86 amino acids containing a putative signal sequence and a pro-region. Within this pro-region, a collagenase target sequence is present, suggesting that self-processing is involved in maturation of the collagenase enzyme (Kameyama and Akama, 1971). Removal of the signal peptide and pro-sequence generates a mature extracellular collagenase (116 kDa) composed of a collagen binding module, a polycystic kidney disease-like (PKD-like) domain and a collagenase module, which contains a zinc-binding sequence (HEXXH) at the center of the active site (Matsushita et al., 1994). The *C. perfringens* collagenases have been studied mostly with respect to their role in the pathogenesis of gangrene. Little research, however, has been conducted on the role of the clostridial collagenases in intestinal disease.

Necrotic Enteritis (NE) is an enteric disease of broiler chickens which is caused by avian-specific, NetB toxin producing strains of *C. perfringens* (i.e. toxinotype G strains) (Long et al., 1974; Rood et al., 2018). The secreted pore-forming NetB toxin is a key virulence factor in the development of necrotic ulcers which are typical for NE. Yet, where and how NetB initiates this process is not fully understood (Keyburn et al., 2008; Rood et al., 2016). A recent study focusing on the gene expression during colonization of the chicken intestinal tract by *C. perfringens* showed that NetB expression is not upregulated during the early stages of disease. It is currently believed that the toxin targets deeper layers of the intestinal mucosa rather than superficial structures (Parreira et al., 2016). As a consequence, initial breakdown or permeabilization of the intestinal mucosa might be needed in order for NetB to exert its action. This permeabilization might be caused by a combination of factors such as agents causing epithelial damage [e.g. *Eimeria* spp. (Al-Sheikhly and Al-Saieg, 1980) or mycotoxins (Ren et al., 2019)], either or not in combination with collagenolytic enzymes which could affect the connective tissue and basement membrane in the intestinal mucosal layer, and thus initiate lesions (Olkowski et al., 2006; Olkowski et al., 2008). *C. perfringens* strains isolated from field cases of NE secrete several potent collagenolytic enzymes and the expression of the *colA* gene is upregulated during the early stages of NE (Olkowski et al., 2008; Parreira et al., 2016), suggesting a potential role in the initial stages of the pathogenesis of NE.

The aim of the present study was to elucidate whether kappa toxin (~120 kDa collagenase encoded by *colA*) from avian pathogenic (NetB positive) *C. perfringens* strains (i.e. toxinotype G) differs from that of commensal (NetB negative) chicken-associated *C. perfringens* strains (i.e. toxinotype A) both in its sequence and function.

## Materials and Methods

### Bacterial Strains and Culture Conditions

The *colA* gene sequences of 25 C*. perfringens* strains originating from chickens and 23 strains from non-chicken origin were used in this study (**Table 1**). This collection contains strains which were derived from a range of international locations and host disease statuses. From these strains, a selection of 21 chicken-associated *C. perfringens* strains were cultured, either for DNA extraction or for functionality assays (**Table 1**). *C. perfringens* strains were routinely grown on Columbia agar (Oxoid) plates supplemented with 5% defibrinated sheep blood or in Brain Heart Infusion broth (BHI, Oxoid, Basingstoke, UK) under anaerobic conditions at 37°C.

**Table 1:** Characteristics of C. perfringens strains used in this study.

Strain	Toxinotype	Origin	Geographic location	Host disease state	*colA* sequencederived from	*colA type^2^*	Activity confirmation^3^	Reference
1207_CPER	Type A	Human	Washington, USA	Intensive care unit patient	JVYV00000000	Full-length		Roach et al., 2015
4928STDY7387940	Unknown	Human	United Kingdom	Unknown	LR607374.1	Full-length		Biosample database at NCBI, 2019a
AN68	Type A	Chicken	Czech Republic	Healthy	NFHR00000000	Full-length		Medvecky et al., 2018
ATCC_13124	Type A	Human	n/a	Gas gangrene	CP000246	Full-length		Möllby et al., 1976
CP15	Type A	Chicken	Delaware, USA	Healthy	CP019468.1	Full-length		Li et al., 2020
CP23	Type G	Chicken	Belgium	Healthy	MW393536^1^	Type II	X	Gholamiandekhordi et al., 2006
CP24	Type A	Chicken	Belgium	Healthy	MW393539^1^	Full-length	X	Gholamiandekhordi et al., 2006
CP43	Type A	Chicken	Belgium	Necrotic enteritis	MW393540^1^	Full-length	X	Gholamiandekhordi et al., 2006
CP56	Type G	Chicken	Belgium	Necrotic enteritis	MW393528^1^	Type IA	X	Gholamiandekhordi et al., 2006
CP60	Type G	Chicken	Belgium	Necrotic enteritis	MW393532^1^	Type IB		Gholamiandekhordi et al., 2006
CP61	Type G	Chicken	Belgium	Necrotic enteritis	MW393533^1^	Type IB		Gholamiandekhordi et al., 2006
D13	Type G	Chicken	Denmark	Necrotic enteritis	MW393538^1^	Type III		this study
D15	Type G	Chicken	Denmark	Necrotic enteritis	MW393535^1^	Type IB		this study
D2	Type G	Chicken	Denmark	Necrotic enteritis	MW393534^1^	Type IB	X	this study
D3	Type G	Chicken	Denmark	Necrotic enteritis	MW393527^1^	Full-length		this study
D4	Type A	Chicken	Denmark	Necrotic enteritis	MW393546^1^	Type II		this study
D8	Type G	Chicken	Denmark	Necrotic enteritis	MW393534^1^	Type II	X	this study
Del1	Type G	Chicken	Delaware, USA	Necrotic enteritis	CP019576.1	Type II		Li C. et al., 2017
EHE-NE15	Type G	Chicken	Australia	Necrotic enteritis	MW393530^1^	Type IA		Sheedy et al., 2004
EHE-NE18	Type G	Chicken	Australia	Necrotic enteritis	CP025501.1	Type IA	X	Sheedy et al., 2004
EHE-NE5	Type G	Chicken	Australia	Necrotic enteritis	MW393529^1^	Type IA		Sheedy et al., 2004
F4969	Type A	Human	n/a	Diarrhoea	NZ_ABDX00000000.1	Full-length		Cornillot et al., 1995
FORC003	Type A	Aquarium water	South Korea	n/a	CP009557.1	Full-length		Kiu et al., 2017
JFP727	Type A	Foal	Canada	Necrotizing enteritis	GCA_001949245.1	Full-length		Mehdizadeh Gohari et al., 2016
JFP923	Type A	Dog	USA	Hemorrhagic gastroenteritis	GCA_001949615.1	Full-length		Mehdizadeh Gohari et al., 2016
JFP983	Type A	Foal	USA	Necrotizing enteritis	GCA_001949775.1	Full-length		Mehdizadeh Gohari et al., 2017
JGS1495	Type C	Pig	n/a	Diarrhoea	GCA_000171135.1	Full-length		Fisher et al., 2006
JGS1721	Type D	Sheep	n/a	Enterotoxaemia	NZ_ABOO00000000.1	Full-length		Hassan et al., 2015
JGS1987	Type E	Calf	n/a	Haemorrhagic enteritis	NZ_ABDW00000000.1	Full-length		Lepp et al., 2010
JGS4104	Type A	Chicken	USA	Necrotic enteritis	MW393545^1^	Type II		Cooper and Songer, 2010
JIR4857	Type A	Chicken	Australia	Necrotic enteritis	MW393541^1^	Full-length	X	Van Damme et al., 2020
JJC	Type A	Landfill	Malaysia	n/a	AWRZ01000000	Full-length		Wong et al., 2014
JP55	Type A	Foal	Canada	Necrotizing enteritis	CP010993	Type IV		Mehdizadeh Gohari et al., 2016
JP838	Type A	Dog	Washington, USA	Hemorrhagic gastroenteritis	CP010994.1	Full-length		Mehdizadeh Gohari et al., 2016
JXJA17	Unknown	Pig	China	Unknown	CP028149.1	Full-length		Biosample database at NCBI, 2020
MGYG-HGUT-02372	Unknown	Human	USA	Unknown	LR698985.1	Full-length		Biosample database at NCBI, 2019b
MJR7757A	Type A	Human	n/a	Unknown	NZ_LRPU00000000.1	Full-length		Ribeiro et al., 2012
N11	Type A	Chicken	Maryland, USA	Healthy	CP023410	Full-length		Li C. et al., 2017
NAG-NE24	Type A	Chicken	Australia	Necrotic enteritis	MW393544^1^	Full-length		Sheedy et al., 2004
NCTC 13170	Unknown	Unknown	n/a	Unknown	NZ_LS483393.1	Full-length		Biosample database at NCBI, 2019c
NCTC 2837	Type A	Unknown	n/a	Unknown	NZ_ABDY00000000.1	Full-length		Hauduroy et al., 1937
NCTC 8239	Type A	Human	The Netherlands	Food poisoning	NZ_CABPRS010000001.1	Full-length		Hobbs et al., 1953
PK406	Type A	Chicken	Belgium	Necrotic enteritis	MW393543^1^	Full-length		this study
PM69	Type G	Chicken	Belgium	Necrotic enteritis	MW393531^1^	Type IA		this study
S2	Type A	Chicken	Denmark	Necrotic enteritis	MW393542^1^	Full-length	X	this study
SM101	Type A	Human	n/a	Food poisoning	CP000312.1	Full-length		Li J. et al., 2017
Strain13	Type A	Human	n/a	Gas gangrene	BA000016.3	Full-length		Shimizu et al., 2001
WAL_14572	Type A	Human	n/a	Unknown	NZ_ADLP00000000.1	Full-length		Ribeiro et al., 2012

^1^Sequence derived by sanger sequencing performed in this study as described in materials & methods.

^2^classification of colA types based on the presence of non-sense mutation in the colA gene.

^3^proteolytic activity confirmed by zymography at different stages of bacterial growth.

*Escherichia (E.) coli* TOP10 cells were used as the cloning host for recombinant expression of collagenase and were routinely grown on LB medium (Fisher Scientific, Merelbeke, Belgium) supplemented with 100 μg/ml of ampicillin. Terrific Broth (24% Tryptone, 42% Yeast extract, 4% glycerol, 0.72 M Na_2_HPO_4_, 0.16 M NaH_2_PO_4_) was used for recombinant protein production.

### Growth Curves

Growth curves were performed for a subset of 9 chicken-associated *C. perfringens* strains (**Table 1**). Overnight grown cultures were diluted 1/10,000 in fresh BHI broth and incubated anaerobically at 37°C. Bacterial growth was determined by measuring the optical density at 600 nm (Genesys 10S UV‐vis spectrophotometer, Thermo Scientific, Waltham, MA, USA*)* at 0, 1, 2, 3, 4, 5, 6 and 24 h after inoculation. At each timepoint, 1 mL aliquots from each culture were collected to obtain cell-free supernatants by centrifugation of the samples, followed by filtration of the supernatants through a 0.2 µm filter and stored at -20°C, until further use in zymography (see below).

### Zymography

Supernatant aliquots of 10 µL were mixed with 2 × loading buffer (0.5 M Tris–HCl pH 6.8, 20% glycerol, 4% SDS, a pinch of bromophenol blue) and separated on 8% SDS page containing 0.1% gelatin under non-reducing conditions. After separation, the gel was incubated with renaturing buffer (2.5% Triton X-100, 30 min, room temperature) to remove SDS from the gel. This allows the separated enzymes in the gel to renature and restores enzymatic activity. Subsequently, the gel was washed with developing buffer (150 mM NaCl, 5 mM CaCl_2_, 0.05% NaN_3_ and 50 mM Tris–HCl buffer pH 7.5) and incubated with fresh developing buffer under continuous shaking at 37°C for 18 h. Afterwards, the gel was stained for 1h with Coomassie brilliant blue G-250 (Sigma-Aldrich, Bornem, Belgium) and destained for 20 minutes with destaining solution [40% methanol (v/v), 10% acetic acid (v/v)]. Activity of gelatin-degrading enzymes is visible as clear colourless bands against a blue background. Gels were scanned using a GS-800 calibrated densitometer and the intensities of the gelatinolytic bands were determined using the Quantity One software (BioRad, Hercules, CA, USA). Values were normalized against the background and expressed as arbitrary units $\left(A.U\right)=100*\left(1-\frac{\frac{OD}{mm^{2}}\;of\;sample}{\frac{OD}{mm^{2}}\;of\;background}\right)$

### Assessment of *colA* Sequence Variants

### Retrieval of *colA* Sequences From Published Sequences

EHE-NE18 *colA* sequence was obtained from NCBI and a BLASTn search was performed for *C. perfringens* (tax ID: 1502) genomes across all annotated strains. ColA gene sequences of 28 *C. perfringens* strains (including 5 chicken-associated and 23 non-chicken-associated *C. perfringens strains*) were acquired from the NCBI genome database (**Table 1**).

### PCR Amplification and Sanger Sequencing of the *colA* Genes

**Figure 1:**
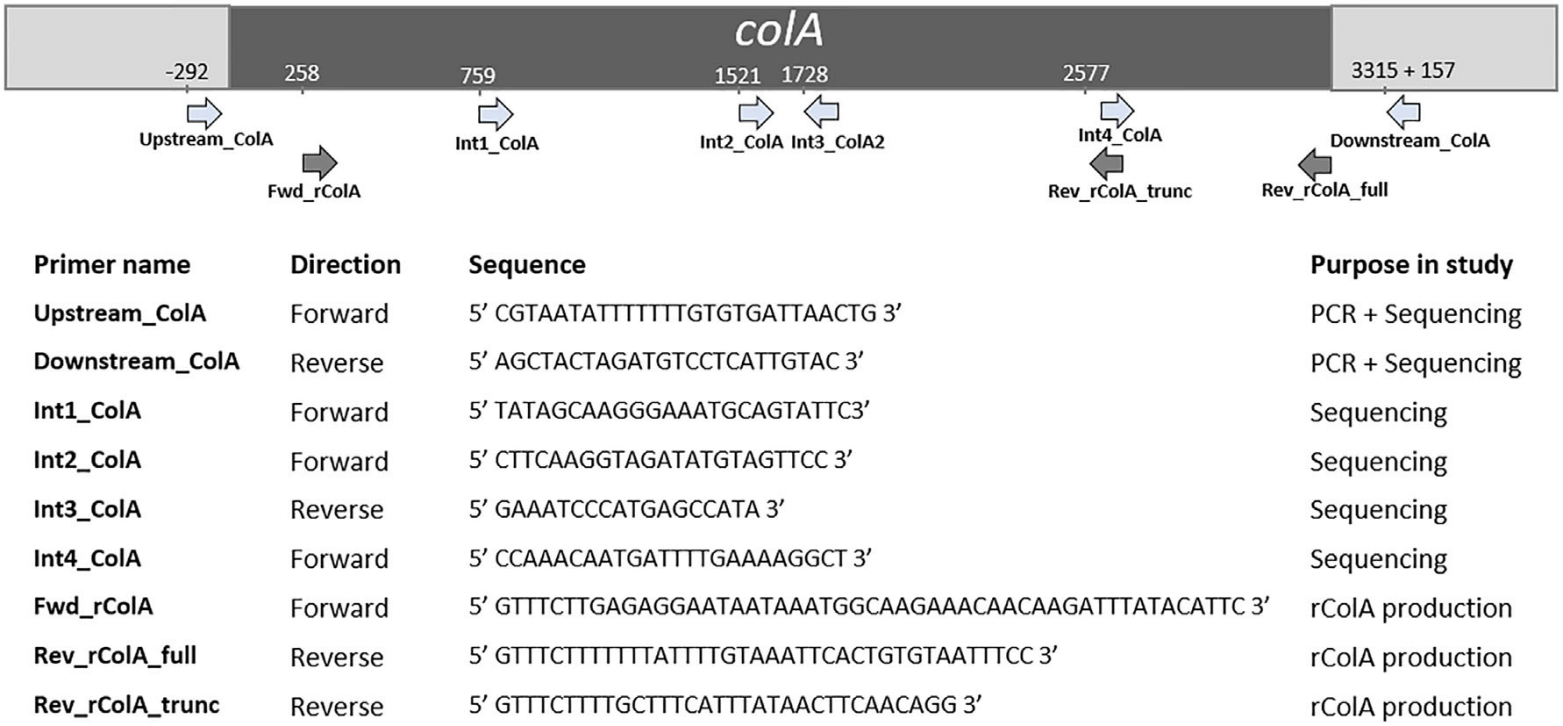
Primers used in this study and their relative position in *colA.* Positions are based on *C. perfringens* strain EHE-NE18 *colA* sequence.

The *colA* gene of a set of 20 chicken-associated *C. perfringens* strains from which no whole genome sequencing data was publicly available, was amplified by PCR and sequenced by Sanger sequencing (**Table 1**). Genomic DNA was extracted using alkaline lysis as previously described (Scheirlinck et al., 2007). Amplification of the *colA* genes was performed in a total reaction volume of 50 μl containing 0.5 µL dNTP Mix (100 mM), 2 μL DNA template, 1 µL Velocity DNA Polymerase, 1× Hi-Fi Buffer (Bioline, London, UK) and 0.4 μM of primers (**Figure 1**). Thermal cycling conditions: initial denaturing step of 98°C for 2 min, followed by 35 amplification cycles of 98°C for 30 s, 50°C for 1 min, and 72°C for 3 minutes and a final extension step of 72°C for 10 min. Presence of a single PCR band was verified by electrophoresis on 1.5% agarose gels. Sanger sequencing was performed by Eurofins Genomics (Ebersberg, Germany). As a single Sanger sequencing run generates a read of up to 1000 basepairs and the *colA* nucleotide sequence is ±3315 bp long, multiple internal sequencing primers were designed to make sure the entire gene sequence was covered. Design of all primers was based on a multiple sequence alignment of the publicly available *colA* sequences. All primers used in this work are shown in **Figure 1**. Nucleotide sequences are available in the GenBank sequence database under accession numbers MW393527 to MW393546.

### Genetic Analysis of the *colA* Genes

The amino acid sequences encoded by the *colA* gene of 48 *C. perfringens* strains (**Table 1**) were deduced bioinformatically using the ExPASy Translate tool (https://web.expasy.org/translate). The domain architecture of the deduced protein fragments was retrieved by performing an NCBI Conserved Domain search and the molecular weights of the mature collagenase fragments (i.e. excluding the pre-pro domains) were calculated using the ExPASY pI/MW tool (https://web.expasy.org/compute_pi). Nucleotide and deduced amino acid sequence data were aligned by the ClustalW alignment algorithm using Mega version X and the alignment was visualized by BOXSHADE (Kumar et al., 2018). Dendrograms of full-length sequences of the *colA* genes (including both fragments when nonsense mutations leading to premature stop codons are present) were constructed by multiple alignment-based similarity coefficients and UPGMA (unweighted pair group method with arithmetic mean) cluster analysis using BioNumerics version 7.6.3 (Applied Maths, Sint-Martens-Latem, Belgium).

### Recombinant Production

Full-length and truncated *colA* gene products were expressed in *E. coli* TOP10 cells using the pBAD TOPO^®^ TA Expression Kit (Invitrogen, Paisley, UK). The desired fragments were amplified from the DNA of *C. perfringens* strains CP56 and CP24 (for respectively the full-length and truncated *colA* gene) by PCR using a DNA polymerase with proofreading activity (Velocity DNA Polymerase, Bioline, Brussels, Belgium). The forward primer (FWD_rColA, **Figure 1**) contained an in-frame stop codon and translation re-initiation sequence to remove the N-terminal leader sequence and allow native protein expression. The reverse primers (REV_rColA_full or REV_rColA_trunc, **Figure 1**) excluded the native *colA* gene stop codon and included the C-terminal V5 epitope and poly-histidine region for affinity purification. Amplifications were performed in a total reaction volume of 50 μl containing 0.5 µL dNTP Mix (100 mM), 2 μL DNA template, 1 µL Velocity DNA Polymerase, 1× Hi-Fi Buffer (Bioline) and 0.4 μM of primers. PCR conditions used for the amplification were an initial denaturation at 95°C for 4 min followed by 35 cycles of denaturation at 95°C for 1.5 min, annealing at 53.8°C for 30 s and extension at 72°C for 2 min, and a final extension step at 72°C for 10 min. The resulting PCR product was incubated with Taq polymerase for 10 min at 72°C (5 U; Promega, Madison, WI, USA) to add 3’ A-overhangs, cloned into the pBAD-TOPO expression vector (Invitrogen, Merelbeke, Belgium), and transformed into One Shot TOP10^®^ *E. coli* (Invitrogen) according to the manufacturer’s instructions. The correct orientation of inserts was verified by Sanger sequencing.

Recombinant *E. coli* carrying the pBAD-collagenase vectors were grown in Terrific Broth supplemented with 100 µg/mL ampicillin at 37°C until an OD_600_ of 0.5 was reached. Expression of the recombinant full-length and truncated rColA was induced overnight with 0.2% L-arabinose (w/v). Bacteria were harvested by centrifugation and lysed enzymatically using BugBuster (Invitrogen, Merelbeke, Belgium). Recombinant proteins were purified on a Ni-sepharose column (His Gravitrap, GE Healthcare Bio-Sciences AB, Uppsala, Sweden) according to the manufacturer’s instructions. Subsequently, proteins were dialyzed against PBS, purity was analysed using SDS-PAGE, and protein concentration was measured using BCA protein assay (Fisher Scientific). Activity of the recombinant proteins was analysed by gelatin zymography (0.5 µg protein load) as described above.

### Gelatinase/Collagenase Activity Assay

The Molecular Probes EnzChek^®^ Gelatinase/Collagenase Assay Kit was used to evaluate the activity of the recombinant collagenase fragments using DQ™ Gelatin (proxy for total collagenolytic activity), DQ™ Collagen I (activity against fibrillar collagen) and DQ™ Collagen IV (activity against non-fibrillar collagen). Different recombinant enzyme concentrations (5 µg/mL, 1 µg/mL or 0.1 µg/mL) were used to avoid effects of enzyme-substrate interactions. In a 96-well format, 50 µL of each recombinant sample was mixed with 130 μL of reaction buffer (0.5 M Tris–HCl, 1.5 M NaCl, 50 mM CaCl_2_ and 2 mM sodium azide at pH 7.6) and 20 µL of either fluorescein labelled substrate (DQ Collagen I (25 μg/mL), DQ Collagen IV (25 μg/mL) or DQ Gelatin (12.5 μg/mL)). Samples without enzyme activity served as control for auto-fluorescence. Reactions were incubated for 60 min at room temperature in the absence of light, and fluorescence was measured every 5 min (excitation 485 nm, emission 527 nm; Fluoroskan Ascent Fluorometer, Thermo Fisher Scientific). Activity of the recombinant collagenase fragments against each substrate was calculated through the slope of the linear phase (first 30 minutes) of the fluorescence resulting from the cleavage of the respective fluorescein labelled substrate over time

$$\left(enzyme\;activity=\frac{\left(slope\;enzyme-slope\;blank\right)\;\times \;dilution\;factor}{0,001\;\times \;enzyme\;volume}\right)$$

Subsequently, the activity of each recombinant fragment against collagen type I or IV was normalized against its total collagenolytic activity and the ratio

$$\frac{relative\;enzyme\;activity{\;}_{collagenase\;variant\;I}}{relative\;enzyme\;activity_{full-length\;collagenase}}$$

was calculated. One-sample t-tests were performed to evaluate whether the ratios differed significantly from 1, i.e. the ratio value that corresponds with an equal activity of both collagenase variant I and full-length collagenase. All statistical analyses were performed using GraphPad Prism software (version 5.03, San Diego, CA, USA).

## Results

### Differential Gelatinolytic Profiles Are Observed Between Chicken-Associated Commensal and Pathogenic *C*. *perfringens* Strains

**Figure 2:**
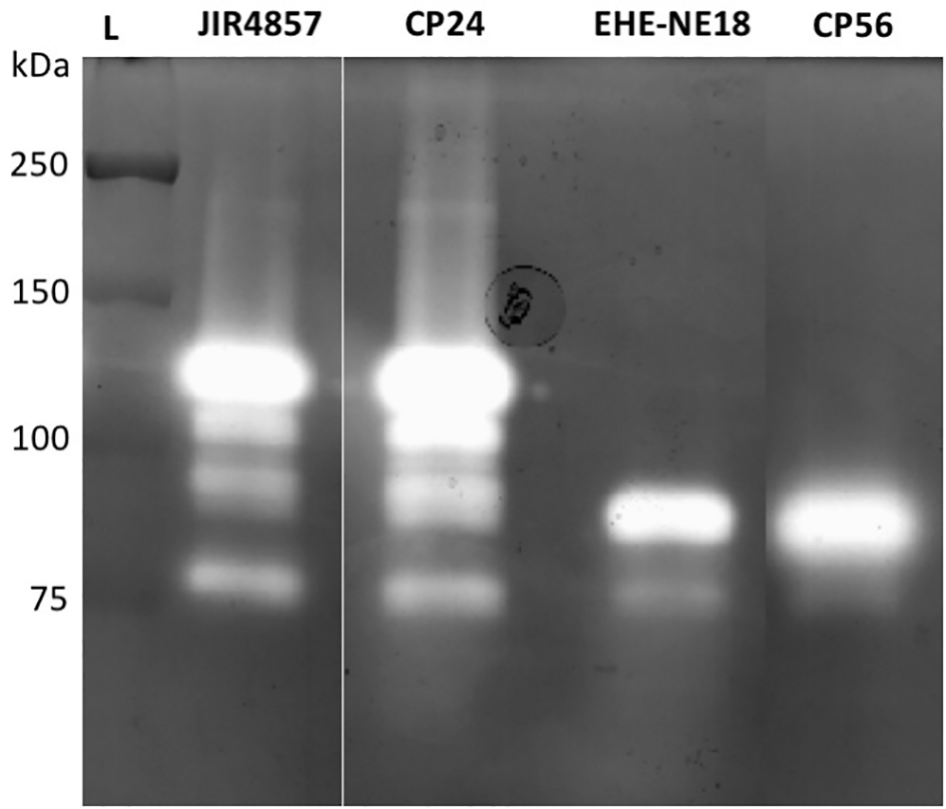
Gelatinolytic activities expressed by different *C. perfringens* strains. Supernatant samples of 2 *C. perfringens* type A strains (JIR4857, CP24) and 2 *C. perfringens* type G strains (EHE-NE18, CP56) were analyzed for proteolytic activity by gelatin zymography. Lane L, Protein Ladder (Precision Plus Protein All Blue Pre-stained Protein Standards, Biorad).

To determine whether pathogenic *C. perfringens* type G strains harbor different collagenolytic properties as compared to commensal *C. perfringens* type A strains, the *in vitro* collagenase production of two type G strains with proven pathogenic activity (CP56 and EHE-NE18) was compared to two commensal *C. perfringens* type A isolates (JIR4857; proven to be non-pathogenic and CP24; isolated from a healthy chicken) (Gholamiandekhordi et al., 2006; Gharib-Naseri et al., 2019; Van Damme et al., 2020). Both *C. perfringens* type A strains showed 1 major band of activity at ± 116 kDa and 3 minor bands with molecular masses between 75 and 110 kDa. Remarkably, a different pattern was seen for the *C. perfringens* type G strains. For both of these strains a major band of molecular size 80-90 kDa and a fainter band of ±75 kDa was observed (**Figure 2**). The 116 kDa collagenase band, which is the predicted height of the *colA* gene product was absent in both tested pathogenic type G strains.

### Truncated Collagenase Variants Are More Frequently Found in Chicken *C*. *perfringens* Type G Strains

**Figure 3:**
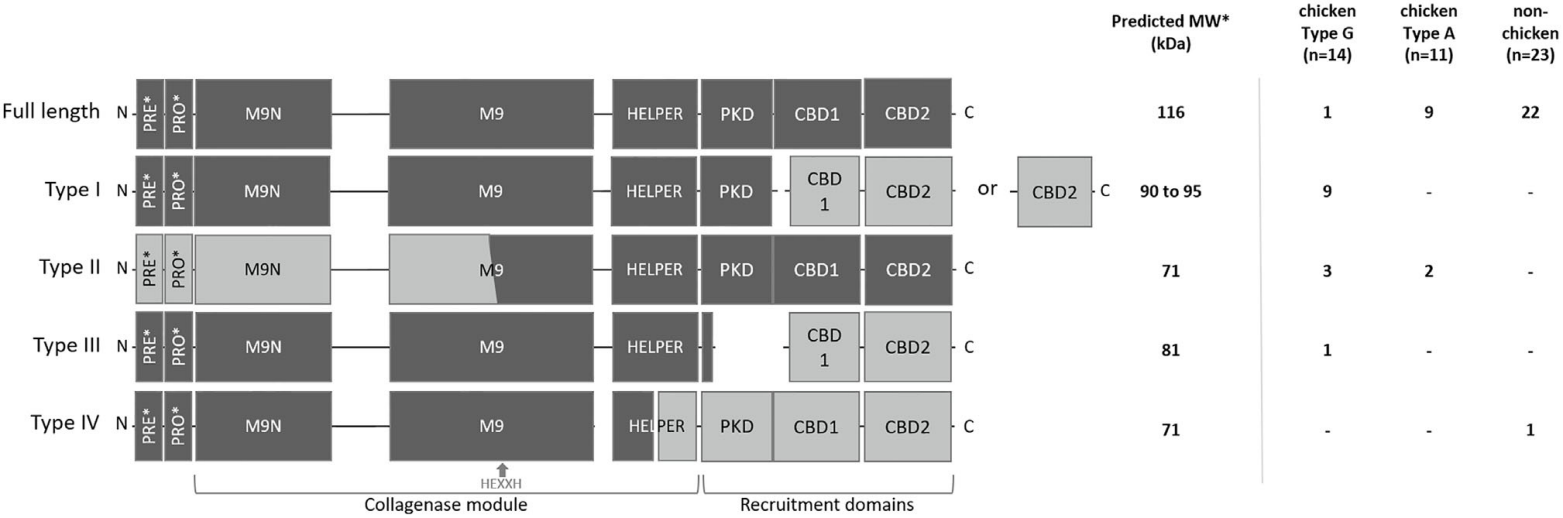
Schematic representation and distribution of collagenase variants. Schematic representation of the domain architecture of full-length and truncated collagenase types and their distribution among chicken (type G and type A strains) and non-chicken derived *C. perfringens* strains (respectively n=14, n=11 and n=23). PRE = signal sequence, PRO = pro-domain, M9N = activator domain, M9 = peptidase domain, HELPER = peptidase helper domain, PKD = polycystic kidney disease-like domain and CBD = collagen binding domains. Collagenase variant types are encoded by gene variants carrying a premature stop mutation in the colA gene which results in the occurrence of 2 protein-coding regions. Predicted active fragment containing the HEXXH motif is represented in dark grey, while the other fragment which is expected to be inactive is represented in light grey. *PRE & PRO domains are removed upon maturation. Molecular weight of the predicted mature active fragment is given.

To explain the appearance of these different gelatinolytic profiles, the sequences of the *colA* gene of a collection of 48 C*. perfringens* strains were compared. Overall, the *colA* sequences were highly similar at nucleic acid level (> 96.6% sequence identity). Variations due to deletions, insertions and single-nucleotide polymorphisms were observed in 259 out of the 3316 nucleotide positions. Comparative analysis of the deduced amino acid sequences demonstrated that there were multiple variants containing nonsense mutations leading to the expression of truncated collagenase fragments (**Supplementary Figure S1**). Genetic variants were classified in different collagenase variant types (I to IV) based on the location of the nonsense mutation and the predicted protein domain structure of the corresponding protein fragments (**Figure 3** and **Table 1**).

The full-length collagenase is synthesized as a multimodular pre-pro-protein (predicted MW ~ 126 kDa) which is subsequently processed to its mature extracellular form (~ 116 kDa). This mature form consists of a collagenase module (containing an M9N activator domain, an M9 peptidase domain with an HEXXH zinc-binding motif and a helper domain), a polycystic kidney disease-like (PKD-like) domain and 2 collagen-binding domains (CBD1 and CBD2) (Kameyama and Akama, 1971). Full-length collagenase sequences were present in 95.65% of the non-chicken derived *C. perfringens* strains and 81.82% of the chicken-derived *C. perfringens* type A strains. By contrast, only 7.14% of the chicken-derived *C. perfringens* type G strains encode the full-length protein (**Figure 3**).

All truncated collagenase variant types are encoded by gene variants carrying a premature stop mutation in the *colA* gene which results in the occurrence of 2 coding sequences. For each of these variant types, the first coding sequence corresponds to the truncated protein at the N-terminal part of the full-length protein, which encodes a signal-sequence, while the second coding sequence corresponds to the truncated protein at the C-terminal part of the full-length protein, which lacks any secretion signal (**Figure 3**). As a consequence, only the fragment encoded by the first coding sequence will be secreted. For collagenase variant type I, this results in an active secreted fragment that contains a complete collagenase module and PKD domain yet lacks the 2 collagen binding domains. Depending on the location of the premature stop codon, two subtypes (e.g. type IA and IB) can be described which both encode a similar active collagenase protein (type I variant). The main difference between these subtypes is found in the second gene fragment, which encodes either both CBD domains (variant type IA) or only a single CBD domain (variant type IB). Both type I variants are exclusively present in *C. perfringens* type G strains (respectively present in 35.7% and 28.6% of the type G strains) (**Figure 3**).

Collagenase type II variants were present in 18.2% of the chicken-derived *C. perfringens* type A strains and 21.4% of the type G strains and were absent in non-chicken derived *C. perfringens* strains. Unlike all other collagenase variant types, the 2 coding sequences of collagenase type II variants are located on overlapping open reading frames. As a result, the M9 peptidase-domain is encoded in part by the first and in part by the second coding sequence. Only the second fragment is predicted to be active as it contains the conserved HEXXH* *motif which is characteristic for the catalytic center of a metalloproteinase. Besides a partial, HEXXH-containing M9 peptidase domain, this fragment also contains the collagenase helper domain, the PKD-like domain and 2 CBDs, yet it lacks secretion signal sequence, indicating that the protein cannot be secreted by the bacteria (**Figure 3**).

The third collagenase variant type (= type III) is uniquely present in one *C. perfringens* type G strain. In this type, the longest sequence encodes a fragment containing the pre-pro-region, the collagenase module and a small part of the PKD-like domain, while the other coding sequence only encodes the domains which are responsible for collagen binding.

In contrast to the collagenase variant types I, II and III, which were exclusively found in chicken-derived *C. perfringens* strains, the fourth collagenase variant type (type IV) was unique for one non-chicken derived *C. perfringens* strain. The *colA* gene of the collagenase type IV variant encodes a first fragment containing the pre-pro-region and a part of the collagenase module (i.e. half of the peptidase helper domain is missing) and a second fragment consisting of the other half of the helper domain, the PKD-like domain and both CBDs. The former fragment is expected to be active upon maturation (**Figure 3**).

### Phylogenetic Relationships of the Collagenase Variants

**Figure 4:**
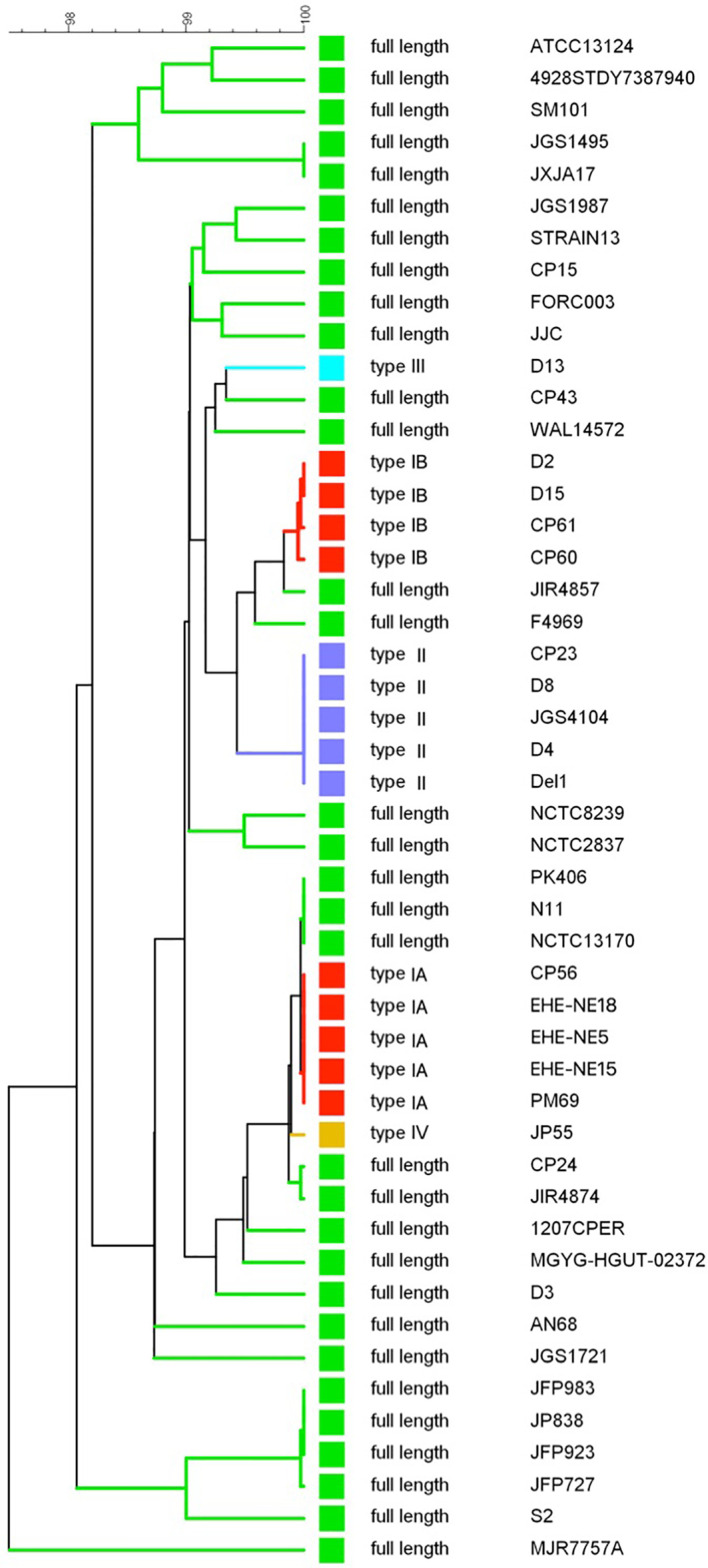
*colA* gene based dendrogram reflecting the similarity for 48 *C. perfringens* strains. A UPGMA phylogenetic tree was constructed based on the *colA* gene sequences (i.e. full gene sequences - from original start to original stop codon - including both fragments when nonsense mutations leading to premature stop codons are present). Sequence similarity percentages (top bar) are shown. Colored branches depict collagenase variant types (see **Figure 3**, Green, full-length; Red, collagenase type I variant; Blue, collagenase type II variant; Light blue, collagenase type III variant; Orange, collagenase type IV variant). Branch labels include the *C. perfringens* strains from which the *colA* gene sequence was derived.

A UPGMA phylogenetic tree was constructed based on the *colA* gene sequences (i.e. full gene sequence including both fragments when nonsense mutations leading to premature stop codons are present) of a collection of 48 C*. perfringens* strains (**Figure 4**). In general, *C. perfringens* strains encoding the same collagenase variant type clustered together showing very similar (e.g. type IB) or even identical (e.g. type IA or type II) nucleotide sequences. In contrast, *C. perfringens* strains encoding the full-length collagenase were found more scattered throughout the phylogeny and branch at various depths in the phylogeny, reflecting a higher diversity between these strains.

### Activity Confirmation of the Different Collagenase Variant Types

**Figure 5:**
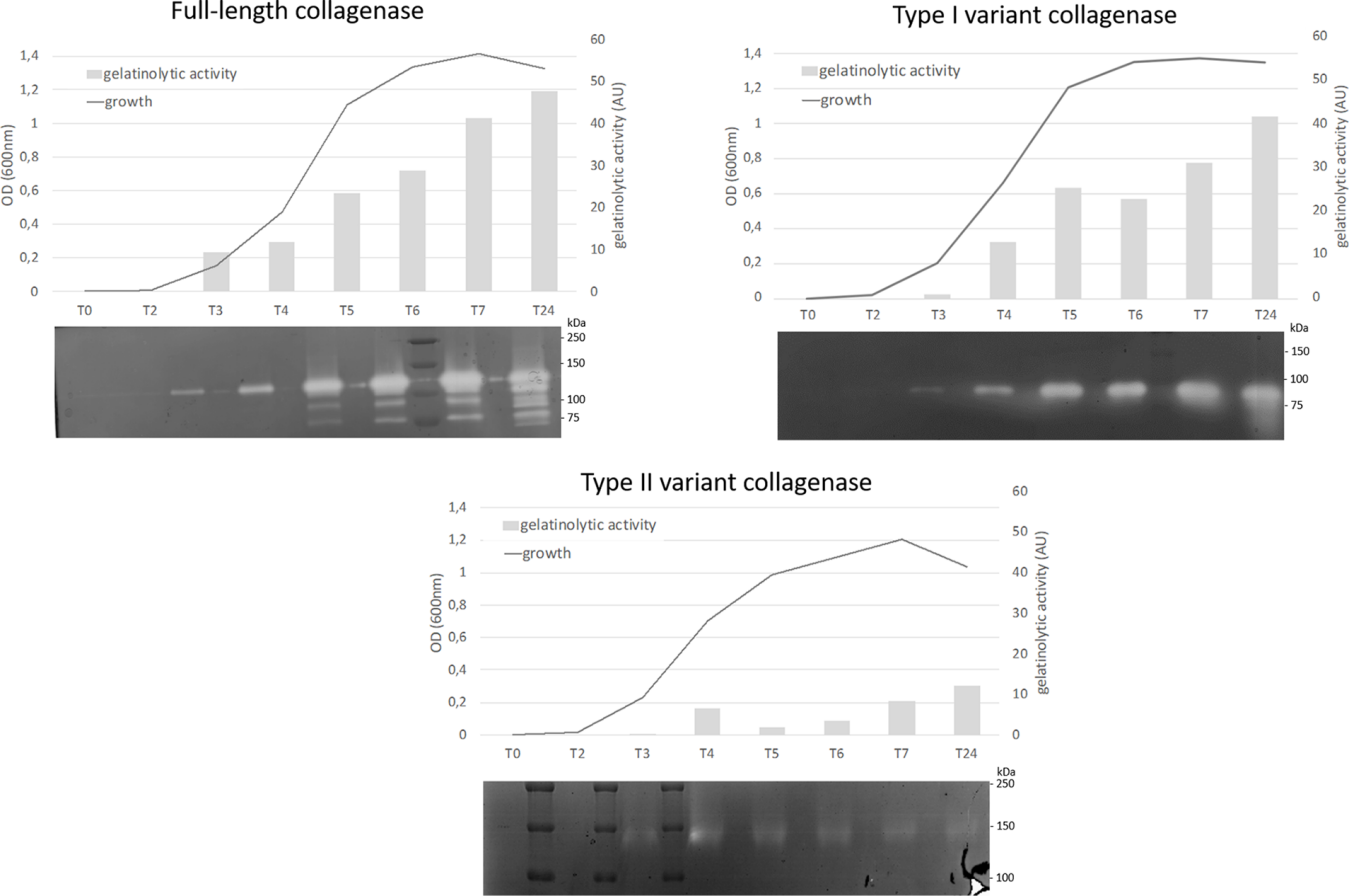
Analysis of proteolytic activity at different stages of growth of *C. perfringens* strains encoding for 3 different collagenase types. Growth was analyzed by measuring optical density at 600 nm at 8 timepoints (0, 2, 3, 4, 5, 6, 7 and 24 h post inoculation). At each timepoint supernatant samples were collected and analyzed by zymography. The observed bands were measured by Quantity One to determine the relative gelatinolytic activity. AU = arbitrary units. One representative image per collagenase variant type is shown (full-length collagenase: JIR4857, type I: CP56 and type II: D8).

All collagenase variant types were predicted to be catalytically active as they all contain an intact HEFTH (HEXXH) motif (**Supplementary Figure S1**). In order to verify this, a zymographic analysis was performed on supernatant samples of 9 C*. perfringens* strains encoding for the most common collagenase types (*i.e.* full-length collagenase (n=4), variant type I (n=2 for subtype IA and n=1 for subtype IB) and variant type II strains (n=2) (**Table 1** and **Figure 5**).

All strains encoding the full-length collagenase showed proteolytic activity at the predicted molecular weight of 116 kDa in the early log phase. Later in growth, several catalytically active fragments with molecular weights ranging from 116 to approximately 80 kDa were observed. Likewise, strains encoding the collagenase type I variants, showed a single band of activity at the expected height (~ 90 kDa or ~ 95 kDa) early in growth, while on later time points one or more additional lower molecular weight band(s) (<75 kDa) became visible. In one strain, an additional band of higher molecular weight (90-110 kDa) was observed (strain S2, data not shown).

Type II variant collagenases lack a signaling peptide needed for protein secretion (described above), resulting in either no gelatinolytic activity observed in the supernatants (strain CP23, data not shown) or one very faint band of ± 140 kDa, which corresponds to the molecular weight of the dimeric form of the mature variant II collagenase fragment (monomer ~ 71 kDa) and might be released in the supernatants as the result of release of intracellular proteins from disrupted dead bacteria.

### Both Recombinant Full-Length and Variant Type I Collagenase Have Active Auto-Cleavage Products

**Figure 6:**
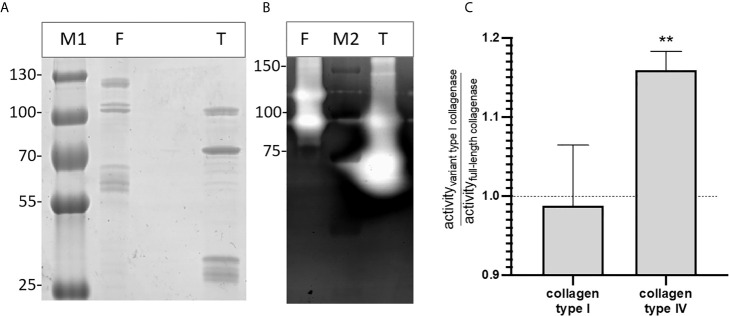
Recombinant full-length and variant type I collagenase have active auto-cleavage products. **(A)** SDS page and **(B)** Gelatin zymography of the recombinantly expressed collagenase fragments. F = full-length collagenase, expected fragment 120 kDa; T = Type I variant collagenase, expected fragment 94 kDa; M1= Pageruler plus pre-stained, M2 = Precision Plus protein All blue Pre-stained Protein standard. **(C)** Substrate-degrading activity of both the full-length collagenase and variant type I collagenase towards collagen type I or collagen type IV was measured using the EnzChek collagenase assay. The bar shows the activity of the variant type I collagenase relative to the full-length collagenase towards each of the tested substrates. Data represents mean ± standard deviations (n=3). One-sample t-tests were performed to evaluate whether the ratios differed significantly from 1, i.e. the ratio value that corresponds with an equal activity of both collagenase variant I and full-length collagenase. **p ≤ 0,01.

Gelatin zymography confirmed that both the full-length and type I recombinant collagenase fragments have gelatinolytic activity (**Figure 6**). Both proteins showed activity at the predicted height together with an active fragment at a lower molecular weight. For the full-length recombinant collagenase, both observed bands (~120 kDa and ~90-100 kDa) accounted for approximately half of the gelatinolytic activity (respectively ± 49% and ± 51%). For the type I recombinant collagenase, the predicted fragment (~94 kDa), only accounted for 26% of the activity, while the breakdown product (~75 kDa) accounted for 74% of the gelatinolytic activity, even though both fragments accounted for similar portions (respectively 27 and 33%) of the total protein detected on SDS-page.

The relative enzyme activity of recombinant full-length and truncated variant type I collagenase against interstitial (type I) and non-fibrillar (type IV) collagen was determined. Both recombinant fragments were capable of degrading type I and type IV collagen. Moreover, the variant type I collagenase showed a higher relative activity against collagen type IV as compared to full-length collagenase (t=11.69, df=2, p=0,0072), whereas no statistically significant difference in activity was seen against collagen type I (t=0.2750, df=2, p=0,8091) (**Figure 6C**).

## Discussion

Bacterial collagenases are enzymes involved in the degradation of the extracellular matrix due to their ability to digest native collagen. These enzymes might play a role in *C. perfringens-*induced NE by facilitating toxin entry in the intestinal mucosa and by destruction of the tissue structure (Kalisz, 1988; Harrington, 1996; Singh et al., 2012; Tomlin and Piccinini, 2018). In the past, *C. perfringens* has been shown to produce various gelatinolytic enzymes with molecular masses ranging from approximately 120 to approximately 80 kDa. Yet, only one gene (*colA*) encoding the 120-kDa collagenase was identified and characterized (Kameyama and Akama, 1971).

In this study, several gene variants carrying different nonsense mutations in the *colA* gene were identified which were mainly linked to *C. perfringens* type G strains, that are pathogenic for broiler chickens. Indeed, although the *colA* gene showed a high level of conservation (> 96% nucleotide sequence identity), truncating variants were identified encoding four different collagenase variant types (type I to IV). These truncated collagenase variants were detected in 13 out of the 14 investigated *C. perfringens* type G strains, which originated from four different countries, on three different continents (USA, Europe and Australia). This indicates that the observed association of truncated collagenase versions with Type G strains is widespread and not due to single mutation event. The most prevalent collagenase variant types were type I (9/48) and II (5/48), whereas both collagenase type III and IV were present in only 1 of the analyzed *C. perfringens* strains. All collagenase variant types were predicted to be active as they all contain an intact HEXXH motif which is characteristic for the catalytic center of a metalloproteinase. However, little to no gelatinolytic activity was observed in the supernatant of strains encoding type II variant collagenases. This might be due to the fact that this collagenase type lacks a signaling peptide needed for protein secretion, or because it is inefficiently transcribed. The active fragments of collagenase variant types I, III and IV have a (nearly) complete collagenase module but lack parts of the recruitment domains (i.e. type I and III miss the CBD domains, type IV misses both PKD and CBD domains). Studies using the ColG collagenase from *Clostridium histolyticum* showed that these recruitment domains are needed to degrade dense collagen networks, such as fibrils, but are not required for the degradation of triple helical collagen (Eckhard and Brandstetter, 2011; Eckhard et al., 2011). However, in this study we showed that recombinant variant type I collagenase - which misses the CBD domains - was able to degrade both interstitial (collagen type I) and non-fibrillar (collagen type IV) collagens. It also had a higher relative activity against collagen type IV as compared to recombinant full-length collagenase. Type IV collagen is the major structural scaffold of basement membranes (Sand et al., 2019). Consequently, these truncated collagenases might be able to break down collagen type IV in the epithelial basement membrane of the intestinal villi. As such, they can contribute to the pathogenesis of NE, as the process leading to intestinal mucosal necrosis has been shown to start at the level of the basement membrane and the lateral domain of the enterocytes (Olkowski et al., 2006; Olkowski et al., 2008). Moreover, the loss of the collagen binding domains in these collagenase variants might allow them to more freely and transiently interact with collagens in the extracellular matrix, potentially leading to a higher non-specific activity resulting in an undefined breakdown of the ECM surrounding the cells, and so contributing to the pathogenesis. Even more, epithelial damage because of predisposing factors such as *Eimeria* might cause easy accessibility to structures underlying the epithelial cells, so that these collagenases can cause further damage to the initial lesion.

Based on the results of our genetic analysis, we showed that genes encoding for a truncated collagenase are more frequently found in pathogenic *C. perfringens* type G strains as compared to commensal chicken-derived *C. perfringens* type A strains or strains originating from other host species, which indicates that having a smaller collagenase might provide an advantage for the bacteria. Indeed, using recombinant collagenase fragments, we showed that both the full-length and variant type I collagenase undergo* *(auto)proteolysis which results in smaller, but still active collagenase fragments. Additionally, we showed that the cleavage product of the type I collagenase accounts for more of its gelatinolytic activity than the original fragment, despite both fragments being present in approximately equal amounts. It is plausible that these smaller collagenases (e.g. truncated variants) might more efficiently migrate in the mucus layer and/or host tissue. This is an issue for future work to explore.

## Data Availability Statement

The datasets presented in this study can be found in online repositories. The names of the repository/repositories and accession number(s) can be found in the article/**Supplementary Material**.

## Authors Contributions

Study design: LV, RD, FV, EG, MD, MF, SH, FT, and SP. Bioinformatic analysis: EG and LV. *In vitro* experiments: LV, NC, and CC. Preparation of the manuscript: LV, FH, RD, FV, and EG. All authors offered a critical review of the manuscript. All authors contributed to the article and approved the submitted version.

## Funding

EG is supported by the Research Foundation Flanders (FWO) under grant number [12W8919N]. The authors declare that this study received funding from Evonik Operations GmbH, Division Nutrition & Care. The funder had the following involvement in the study: Development of the study design. The funder was not involved in the collection, analysis and interpretation of the data or the writing of the article.

## Conflict of Interest

Authors MD, MF, SH, FT, and SP were employed by the company Evonik Operations GmbH.

The remaining authors declare that the research was conducted in the absence of any commercial or financial relationships that could be construed as a potential conflict of interest.

## Abbreviations

NE, necrotic enteritis; *C. perfringens, Clostridium perfringens;* ECM, extracellular matrix.
