# Supplementary material for: Protein Truncating Variants of colA in Clostridium perfringens Type G Strains
Source: Front Cell Infect Microbiol. 2021 Apr 29;11:645248. doi: 10.3389/fcimb.2021.645248 (PMC8117337; doi:10.3389/fcimb.2021.645248)
Supplement: Supplementary file 1 [file DataSheet_1.pdf]

Figure S1

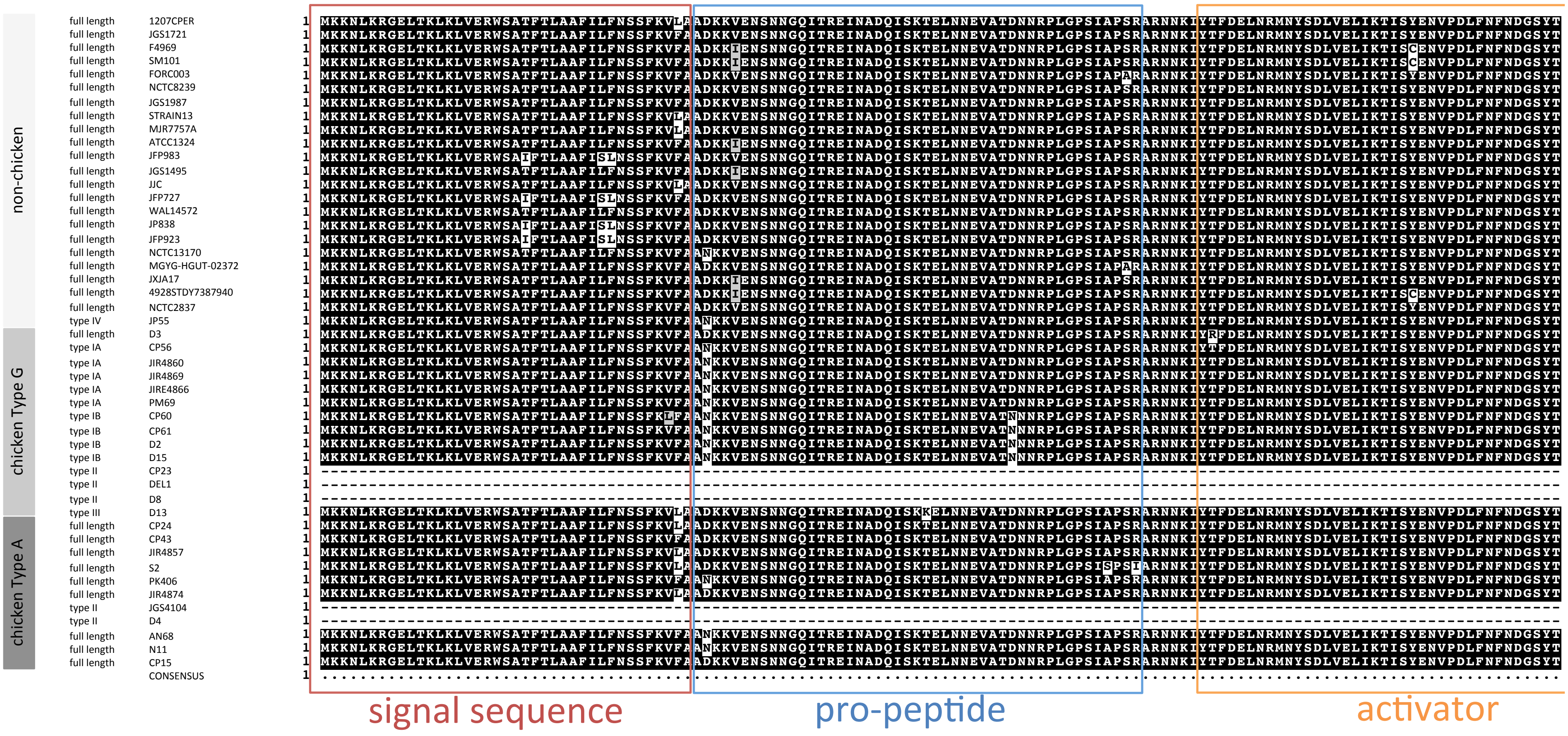

S1 Alignment of the deduced amino acid sequence of the predicted active collagenase fragments of 48 *C. perfringens* strains.

Sequences were aligned using ClustalW and shaded by BOXSHADE. Numbers to the left of sequences indicate the amino acid residue locations. Black shading indicates that the residue is identical to the column consensus; no shading indicates that the residue is different from the consensus; dashes indicate gaps for best alignment. Signal sequence, pro-region, collagenase module (including activator, peptidase and helper domain), PKD-like domain and CBDs are framed. Collagenase sequences were classified in different collagenase types (full-length or variant type I to IV; described in this study) based on the occurrence of the non-sense mutations and the predicted protein domain structure of the corresponding protein fragments.

Figure S1 (cont.)

non-chicken | chicken Type G | chicken Type A

full length	1207CPER	131	FFSNRDRVQAIIYGLEDSGRTYTADDDKGIPTLVEFLRAGYYLGFYNKQLSYLNTPQLKNECLPAMKAIQYNSNFRLGTKAQDGVVEALGRLIGNASADPEVINNCIYVLSDFKDNIDKYGSNYSKGNAV
full length	JGS1721	131	FFSNRDRVQAIIYGLEDSGRTYTADDDKGIPTLVEFLRAGYYLGFYNKQLSYLNTPQLKNECLPAMKAIQYNSNFRLGTKAQDGVVEALGRLIGNASADPEVINNCIYVLSDFKDNIDNYGSNYSKGNAV
full length	F4969	131	FFSNRDRVQAIIYGLEDSGRTYTADDDKGIPTLVEFLRAGYYLGFYNKQLSYLNTPQLKNECLPAMKAIQYNSNFRLGTKAQDGVVEALGRLIGNASADPEVINNCIYVLSDFKDNIDKYGSNYSKGNAV
full length	SM101	131	FFSNRDRVQAIIYGLEDSGRTYTADDDKGIPTLVEFLRAGYYLGFYNKQLSYLNTPQLKNECLPAMKAIQYNSNFRLGTKAQDGVVEALGRLIGNASADPEVINNCIYVLSDFKDNIDKYGSNYSKGNAV
full length	FORC003	131	FFSNRDRVQAIIYGLEDSGRTYTADDDKGIPTLVEFLRAGYYLGFYNKQLSYLNTPQLKNECLPAMKAIQYNSNFRLGTKAQDGVVEALGRLIGNASADPEVINNCIYVLSDFKDNIDKYGSNYSKGNAV
full length	NCTC8239	131	FFSNRDRVQAIIYGLEDSGRTYTADDDKGIPTLVEFLRAGYYLGFYNKQLSYLNTPQLKNECLPAMKAIQYNSNFRLGTKAQDGVVEALGRLIGNASADPEVINNCIYVLSDFKDNIDKYGSNYSKGNAV
full length	JGS1987	131	FFSNRDRVQAIIYGLEDSGRTYTADDDKGIPTLVEFLRAGYYLGFYNKQLSYLNTPQLKNECLPAMKAIQYNSNFRLGTKAQDGVVEALGRLIGNASADPEVINNCIYVLSDFKDNIDKYGSNYSKGNAV
full length	STRAIN13	131	FFSNRDRVQAIIYGLEDSGRTYTADDDKGIPTLVEFLRAGYYLGFYNKQLSYLNTPQLKNECLPAMKAIQYNSNFRLGTKAQDGVVEALGRLIGNASADPEVINNCIYVLSDFKDNIDKYGSNYSKGNAV
full length	MJR7757A	131	FFSNRDRVQAIIYGLEDSGRTYTADDDKGIPTLVEFLRAGYYLGFYNKQLSYLNTPQLKNECLPAMKAIQYNSNFRLGTKEQDGVIEALGRLIGNTSADPEVINNCIYVLSDFKDNIDQYGSNYSKGNAV
full length	ATCC1324	131	FFSNRDRVQAIIYGLEDSGRTYTADDDKGIPTLVEFLRAGYYLGFYNKQLSYLNTPQLKNECLPAMKAIQYNSNFRLGTKAQDGVVEALGRLIGNASADPEVINNCIYVLSDFKDNIDKYGSNYSKGNAV
full length	JFP983	131	FFSNRDRVQAIIYGLEDSGRTYTADDDKGIPTLVEFLRAGYYLGFYNKQLSYLNTPQLKNECLPAMKAIQYNSNFRLGTKAQDGVIEALGRLIGNASADPEVINNCIYVLSDFKDNIDKYGSNYSKGNAV
full length	JGS1495	131	FFSNRDRVQAIIYGLEDSGRTYTADDDKGIPTLVEFLRAGYYLGFYNKQLSYLNTPQLKNECLPAMKAIQYNSNFRLGTKAQDGVVETLGRLIGNASADTEVINNCIYVLSDFKDNIDKYGSNYSKGNAV
full length	JJC	131	FFSNRDRVQAIIYGLEDSGRTYTADDDKGIPTLVEFLRAGYYLGFYNKQLSYLNTPQLKNECLPAMKAIQYNSNFRLGTKAQDGVVEALGRLIGNASADPEVINNCIYVLSDFKDNIDKYGSNYSKGNAV
full length	JFP727	131	FFSNRDRVQAIIYGLEDSGRTYTADDDKGIPTLVEFLRAGYYLGFYNKQLSYLNTPQLKNECLPAMKAIQYNSNFRLGTKAQDGVIEALGRLIGNASADPEVINNCIYVLSDFKDNIDKYGSNYSKGNAV
full length	WAL14572	131	FFSNRDRVQAIIYGLEDSGRTYTADDDKGIPTLVEFLRAGYYLGFYNKQLSYLNTPQLKNECLPAMKAIQYNSNFRLGTKAQDGVVEALGRLIGNASADPEVINNCIYVLSDFKDNIDKYGSNYSKGNAV
full length	JP838	131	FFSNRDRVQAIIYGLEDSGRTYTADDDKGIPTLVEFLRAGYYLGFYNKQLSYLNTPQLKNECLPAMKAIQYNSNFRLGTKAQDGVIEALGRLIGNASADPEVINNCIYVLSDFKDNIDKYGSNYSKGNAV
full length	JFP923	131	FFSNRDRVQAIIYGLEDSGRTYTADDDKGIPTLVEFLRAGYYLGFYNKQLSYLNTPQLKNECLPAMKAIQYNSNFRLGTKAQDGVIEALGRLIGNASADPEVINNCIYVLSDFKDNIDKYGSNYSKGNAV
full length	NCTC13170	131	FFSNRDRVQAIIYGLEDSGRTYTADDDKGIPTLVEFLRAGYYLGFYNKQLSYLNTPQLKNECLPAMKAIQYNSNFRLGTKAQDGVVEALGRLIGNASADPEVINNCIYVLSDFKDNIDKYGSNYSKGNAV
full length	MGYG-HGUT-02372	131	FFSNRDRVQAIIYGLEDSGRTYTADDDKGIPTLVEFLRAGYYLGFYNKQLSYLNTPQLKNECLPAMKAIQYNSNFRLGTKAQDGVVEALGRLIGNASADPEVINNCIYVLSDFKDNIDKYGSNYSKGNAV
full length	JXJA17	131	FFSNRDRVQAIIYGLEDSGRTYTADDDKGIPTLVEFLRAGYYLGFYNKQLSYLNTPQLKNECLPAMKAIQYNSNFRLGTKAQDGVVETLGRLIGNASADTEVINNCIYVLSDFKDNIDKYGSNYSKGNAV
full length	4928STDY7387940	131	FFSNRDRVQAIIYGLEDSGRTYTADDDKGIPTLVEFLRAGYYLGFYNKQLSYLNTPQLKNECLPAMKAIQYNSNFRLGTKAQDGVVEALGRLIGNASADPEVINNCIYVLSDFKDNIDKYGSNYSKGNAV
full length	NCTC2837	131	FFSNRDRVQAIIYGLEDSGRTYTADDDKGIPTLVEFLRAGYYLGFYNKQLSYLNTPQLKNECLPAMKAIQYNSNFRLGTKAQDGVVEALGRLIGNASADPEVINNCIYVLSDFKDNIDKYGSNYSKGNAV
type IV	JP55	131	FFSNRDRVQAIIYGLEDSGRTYTADDDKGIPTLVEFLRAGYYLGFYNKQLSYLNTPQLKNECLPAMKAIQYNSNFRLGTKAQDGVVEALGRLIGNASADPEVINNCIYVLSDFKDNIDKYGSNYSKGNAV
full length	D3	131	FFSNRDRVQAIIYGLEDSGRTYTADDDKGIPTLVEFLRAGYYLGFYNKQLSYLNTPQLKNECLPAMKAIQYNSNFRLGTKAQDGVVEALGRLIGNASADPEVINNCIYVLSDFKDNIDKYGSNYSKGNAV
type IA	CP56	131	FFSNRDRVQAIIYGLEDSGRTYTADDDKGIPTLVEFLRAGYYLGFYNKQLSYLNTPQLKNECLPAMKAIQYNSNFRLGTKAQDGVVEALGRLIGNASADPEVINNCIYVLSDFKDNIDKYGSNYSKGNAV
type IA	JIR4860	131	FFSNRDRVQAIIYGLEDSGRTYTADDDKGIPTLVEFLRAGYYLGFYNKQLSYLNTPQLKNECLPAMKAIQYNSNFRLGTKAQDGVVEALGRLIGNASADPEVINNCIYVLSDFKDNIDKYGSNYSKGNAV
type IA	JIR4869	131	FFSNRDRVQAIIYGLEDSGRTYTADDDKGIPTLVEFLRAGYYLGFYNKQLSYLNTPQLKNECLPAMKAIQYNSNFRLGTKAQDGVVEALGRLIGNASADPEVINNCIYVLSDFKDNIDKYGSNYSKGNAV
type IA	JIRE4866	131	FFSNRDRVQAIIYGLEDSGRTYTADDDKGIPTLVEFLRAGYYLGFYNKQLSYLNTPQLKNECLPAMKAIQYNSNFRLGTKAQDGVVEALGRLIGNASADPEVINNCIYVLSDFKDNIDKYGSNYSKGNAV
type IA	PM69	131	FFSNRDRVQAIIYGLEDSGRTYTADDDKGIPTLVEFLRAGYYLGFYNKQLSYLNTPQLKNECLPAMKAIQYNSNFRLGTKAQDGVVEALGRLIGNASADPEVINNCIYVLSDFKDNIDKYGSNYSKGNAV
type IB	CP60	131	FFSNRERVQAIIYGLEDSGRTYTADDDKGIPTLVEFLRAGYYLGFYNKQLSYLNTPQLKNECLPAMKAIQYNSNFRLGTKAQDGVVEALGRLIGNASADPEVINNCIYVLSDFKDNIDKYGSNYSKGNAV
type IB	CP61	131	FFSNRERVQAIIYGLEDSGRTYTADDDKGIPTLVEFLRAGYYLGFYNKQLSYLNTPQLKNECLPAMKAIQYNSNFRLGTKAQDGVVEALGRLIGNASADPEVINNCIYVLSDFKDNIDKYGSNYSKGNAV
type IB	D2	131	FFSNRERVQAIIYGLEDSGRTYTADDDKGIPTLVEFLRAGYYLGFYNKQLSYLNTPQLKNECLPAMKAIQYNSNFRLGTKAQDGVVEALGRLIGNASADPEVINNCIYVLSDFKDNIDKYGSNYSKGNAV
type IB	D15	131	FFSNRERVQAIIYGLEDSGRTYTADDDKGIPTLVEFLRAGYYLGFYNKQLSYLNTPQLKNECLPAMKAIQYNSNFRLGTKAQDGVVEALGRLIGNASADPEVINNCIYVLSDFKDNIDKYGSNYSKGNAV
type II	CP23	1	--
type II	DEL1	1	--
type II	D8	1	--
type III	D13	131	FFSNRDRVQAIIYGLEDSGRTYTADDDKGIPTLVEFLRAGYYLGFYNKQLSYLNTPQLKNECLPAMKAIQYNSNFRLGTKAQDGVVEALGRLIGNASADPEVINNCIYVLSDFKDNIDKYGSNYSKGNAV
full length	CP24	131	FFSNRDRVQAIIYGLEDSGRTYTADDDKGIPTLVEFLRAGYYLGFYNKQLSYLNTPQLKNECLPAMKAIQYNSNFRLGTKAQDGVVEALGRLIGNASADPEVINNCIYVLSDFKDNIDKYGSNYSKGNAV
full length	CP43	131	FFSNRDRVQAIIYGLEDSGRTYTADDDKGIPTLVEFLRAGYYLGFYNKQLSYLNTPQLKNECLPAMKAIQYNSNFRLGTKAQDGVVEALGRLIGNASADPEVINNCIYVLSDFKDNIDKYGSNYSKGNAV
full length	JIR4857	131	FFSNRDRVQAIIYGLEDSGRTYTADDDKGIPTLVEFLRAGYYLGFYNKQLSYLNTPQLKNECLPAMKAIQYNSNFRLGTKAQDGVVEALGRLIGNASADPEVINNCIYVLSDFKDNIDKYGSNYSKGNAV
full length	S2	131	FFSNRDRVQAIIYGLEDSGRTYTADDDKGIPTLVEFLRAGYYLGFYNKQLSYLNTPQLKNECLPAMKAIQYNSNFRLGTKAQDGVIEALGRLIGNASADPEVINNCIYVLSDFKDNIDKYGSNYSKGNAV
full length	PK406	131	FFSNRDRVQAIIYGLEDSGRTYTADDDKGIPTLVEFLRAGYYLGFYNKQLSYLNTPQLKNECLPAMKAIQYNSNFRLGTKAQDGVVEALGRLIGNASADPEVINNCIYVLSDFKDNIDKYGSNYSKGNAV
full length	JIR4874	131	FFSNRDRVQAIIYGLEDSGRTYTADDDKGIPTLVEFLRAGYYLGFYNKQLSYLNTPQLKNECLPAMKAIQYNSNFRLGTKAQDGVVEALGRLIGNASADPEVINNCIYVLSDFKDNIDKYGSNYSKGNAV
type II	JGS4104	1	--
type II	D4	1	--
full length	AN68	131	FFSNRDRVQAIIYGLEDSGRTYTADDDKGIPTLVEFLRAGYYLGFYNKQLSYLNTPQLKNECLPAMKAIQYNSNFRLGTKAQDGVVEALGRLIGNASADLEVINNCIYVLSDFKDNIDKYGSNYSKGNAV
full length	N11	131	FFSNRDRVQAIIYGLEDSGRTYTADDDKGIPTLVEFLRAGYYLGFYNKQLSYLNTPQLKNECLPAMKAIQYNSNFRLGTKAQDGVVEALGRLIGNASADPEVINNCIYVLSDFKDNIDKYGSNYSKGNAV
full length	CP15	131	FFSNRDRVQAIIYGLEDSGRTYTADDDKGIPTLVEFLRAGYYLGFYNKQLSYLNTPQLKNECLPAMKAIQYNSNFRLGTKAQDGVVEALGRLIGNASADPEVINNCIYVLSDFKDNIDKYGSNYSKGNAV
	CONSENSUS	131	....

activator

Figure S1 (cont)

Group	Type	Strain	Start	Sequence
non-chicken	full length	1207CPER	261	FNLMKGIDYYTNSVIYNTKGYDAKNTEFYNRIDPYMERLESLCTIGDKLNNDNAWLVNNALYYTGRMGKFREDPSISQRALERAMKEYPYLSYQYIEAANDLDLNFGGKNSSGNDIDFNKIKADAREKYL
non-chicken	full length	JGS1721	261	FNLMKGIDYYTNSVIYNTKGYDAKNTEFYNRIDPYMERLESLCTIGDKLNNDNAWLVNNALYYTGRMGKFREDPSISQRALERAMKEYPYLSYQYIEAANDLDLNFGGKDSSGNDIDFNKIKADAREKYL
non-chicken	full length	F4969	261	FNLMKGIDYYTNSVIYNTKGYDAKNTEFYNRIDPYMERLESLCTIGDKLNNDNAWLVNNALYYTGRMGKFREDPSISQRALERAMKEYPYLSYQYIEATNDLDLNFGGKNSSGNDIDFNKIKADAREKYL
non-chicken	full length	SM101	261	FNLMKGIDYYTNLVIYNTKGYDAKNTEFYNRIDPYMERLESLCTIGDKLNNDNAWFVNNALYYTGRMGKFREDPSISQRALERAMKEYPYLSYQYIEAANDLDLNFGGKNSSGNDIDFNKIKADAREKYL
non-chicken	full length	FORC003	261	FNLMKGIDYYTNSVIYNTKGYDAKNTEFYNRIDPYMERLESLCTIGDKLNNDNAWLVNNALYYTGRMGKFREDPSISQRALERAMKEYPYLSYQYIEATNDLDLNFGGKNSSGNDIDFNKIKADAREKYL
non-chicken	full length	NCTC8239	261	FNLMKGIDYYTNSVIYNTKGYDAKNTEFYDRIDPYMERLESLCTIGDKLNNDNAWLVNNALYYTGRMGKFREDPSISQRALERAMKEYPYLSYQYIEAANDLDLNFGGKNSSGNDIDFNKIKADAREKYL
non-chicken	full length	JGS1987	261	FNLMKGIDYYTNSVIYNTKGYDAKNTEFYNRIDPYMERLESLCTIGDKLNNDNAWLVNNALYYTGRMGKFREDPSISQRALERAMKEYPYLSYQYIEAANDLDLNFGGKNSSGNDIDFNKIKADAREKYL
non-chicken	full length	STRAIN13	261	FNLMKGIDYYTNSVIYNTKGYDAKNTEFYNRIDPYMERLESLCTIGDKLNNDNAWLVNNALYYTGRMGKFREDPSISQRALERAMKEYPYLSYQYIEAANDLDLNFGGKNSSGNDIDFNKIKADAREKYL
non-chicken	full length	MJR7757A	261	FNLMKGIDYYTNSVIYNTKGYDAKNTEFYNRIDPYMERLESLCTIGDKLNNDNSWLVNNALYYTGRMGKFRKDPSISQEALERAMKEYPYLSYQYIEAANDLDLNFGGKDSSGNDIDFNKIKADAREKYL
non-chicken	full length	ATCC1324	261	FNLMKGIDYYTNSVIYNTKGYDAKNTEFYNRIDPYMERLESLCTIGDKLNNDNAWFVNNALYYTGRMGKFREDPSISQRALERAMKEYPYLSYQYIEAANDLDLNFGGKNSSGNDIDFNKIKADAREKYL
non-chicken	full length	JFP983	261	FNLMKGIDYYTNSVIYNTKGYDAKNTEFYNRIDPYMERLESLCTIGDKLNNDNAWLVNNALYYTGRMGKFREDPSISQEALERAMKEYPYLSYQYIEAANDLDLNFGGKDSSGNDIDFNKIKADAREKYL
non-chicken	full length	JGS1495	261	FNLMKGIDYYTNSVIYNTKGYDAKNTEFYNRIDPYMERLESLCTIGDKLNNDNAWLVNNALYYTGRMSKFREDPSISQRALERAMKEYPYLSYQYIEAANDLDLNFGGKNSSGNDIDFNKIKADAREKYL
non-chicken	full length	JJC	261	FNLMKGIDYYTNSVIYNTKGYDAKNTEFYNRIDPYMERLESLCTIGDKLNNDNAWLVNNALYYTGRMGKFREDPSISQRALERAMKEYPYLSYQYIEATNDLDLNFGGKNSSGNDIDFNKIKADAREKYL
non-chicken	full length	JFP727	261	FNLMKGIDYYTNSVIYNTKGYDAKNTELYNRIDPYMERLESLCTIGDKLNNDNAWLVNNALYYTGRMGKFREDPSISQEALERAMKEYPYLSYQYIEAANDLDLNFGGKDSSGNDIDFNKIKADAREKYL
non-chicken	full length	WAL14572	261	FNLMKGIDYYTNSVIYNTKGYDAKNTEFYNRIDPYMERLESLCTIGDKLNNDNAWLVNNALYYTGRMGKFREDPSISQRALERAMKEYPYLSYQYIEAANDLDLNFGGKNSSGNDIDFNKIKADAREKYL
non-chicken	full length	JP838	261	FNLMKGIDYYTNSVIYNTKGYDAKNTEFYNRIDPYMERLESLCTIGDKLNNDNAWLVNNALYYTGRMGKFREDPSISQEALERAMKEYPYLSYQYIEAANDLDLNFGGKDSSGNDIDFNKIKADAREKYL
non-chicken	full length	JFP923	261	FNLMKGIDYYTNSVIYNTKGYDAKNTEFYNRIDPYMERLESLCTIGDKLNNDNAWLVNNALYYTGRMGKFREDPSISQEALERAMKEYPYLSYQYIEAANDLDLNFGGKDSSGNDIDFNKIKADAREKYL
non-chicken	full length	NCTC13170	261	FNLMKGIDYYTNSVIYNTKGYDAKNTEFYNRIDPYMERLESLCTIGDKLNNDNAWLVNNALYYTGRMGKFREDPSISQRALERAMKEYPYLSYQYIEAANDLDLNFGGKNSSGNDIDFNKIKADAREKYL
non-chicken	full length	MGYG-HGUT-02372	261	FNLMKGIDYYTNSVIYNTKGYDAKNTEFYNRIDPYMERLESLCTIGDKLNNDNAWLVNNALYYTGRMGKFREDPSISQRALERAMKEYPYLSYQYIEAANDLDLNFGGKNSSGNDIDFNKIKADAREKYL
non-chicken	full length	JXJA17	261	FNLMKGIDYYTNSVIYNTKGYDAKNTEFYNRIDPYMERLESLCTIGDKLNNDNAWLVNNALYYTGRMSKFREDPSISQRALERAMKEYPYLSYQYIEAANDLDLNFGGKNSSGNDIDFNKIKADAREKYL
non-chicken	full length	4928STDY7387940	261	FNLMKGIDYYTNSVIYNTTGYDAKNTEFYNRIDPYMERLESLCTIGDKLNNDNAWLVNNALYYTGRMGKFREDPSISQRALERAMKEYPYLSYQYIEAVNDLDLNFGGKNSSGNDIDFNKIKADAREKYL
non-chicken	full length	NCTC2837	261	FNLMKGIDYYTNSVIYNTKGYDAKNTEFYDRIDPYMERLESLCTIGDKLNNDNAWLVNNALYYTGRMGKFREDPSISQRALERAMKEYPYLSYQYIEAANDLDLNFGGKNSSGNDIDFNKIKADAREKYL
chicken Type G	type IV	JP55	261	FNLMKGIDYYTNSVIYNTKGYDAKNTEFYNRIDPYMERLESLCTIGDKLNNDNAWLVNNALYYTGRMGKFREDPSISQRALERAMKEYPYLSYQYIEAANDLDLNFGGKNSSGNDIDFNKIKADAREKYL
chicken Type G	full length	D3	261	FNLMKGIDYYTNSVIYNTKGYDAKNTEFYNRIDPYMERLESLCTIGDKLNNDNAWLVNNALYYTGRMGKFREDPSISQRALERAMKEYPYLSYQYIEAANDLDLNFGGKNSSGNDIDFNKIKADAREKYL
chicken Type G	type IA	CP56	261	FNLMKGIDYYTNSVIYNTKGYDAKNTEFYNRIDPYMERLESLCTIGDKLNNDNAWLVNNALYYTGRMGKFREDPSISQRALERAMKEYPYLSYQYIEAANDLDLNFGGKNSSGNDIDFNKIKADAREKYL
chicken Type G	type IA	JIR4860	261	FNLMKGIDYYTNSVIYNTKGYDAKNTEFYNRIDPYMERLESLCTIGDKLNNDNAWLVNNALYYTGRMGKFREDPSISQRALERAMKEYPYLSYQYIEAANDLDLNFGGKNSSGNDIDFNKIKADAREKYL
chicken Type G	type IA	JIR4869	261	FNLMKGIDYYTNSVIYNTKGYDAKNTEFYNRIDPYMERLESLCTIGDKLNNDNAWLVNNALYYTGRMGKFREDPSISQRALERAMKEYPYLSYQYIEAANDLDLNFGGKNSSGNDIDFNKIKADAREKYL
chicken Type G	type IA	JIRE4866	261	FNLMKGIDYYTNSVIYNTKGYDAKNTEFYNRIDPYMERLESLCTIGDKLNNDNAWLVNNALYYTGRMGKFREDPSISQRALERAMKEYPYLSYQYIEAANDLDLNFGGKNSSGNDIDFNKIKADAREKYL
chicken Type G	type IA	PM69	261	FNLMKGIDYYTNSVIYNTKGYDAKNTEFYNRIDPYMERLESLCTIGDKLNNDNAWLVNNALYYTGRMGKFREDPSISQRALERAMKEYPYLSYQYIEAANDLDLNFGGKNSSGNDIDFNKIKADAREKYL
chicken Type G	type IB	CP60	261	FNLMKGIDYYTNSVIYNTKGYDAKNTEFYNRIDPYMERLESLCTIGDKLNNDNAWLVNNALYYTGRMGKFREDPSISQRALERAMKEYPYLSYQYIEAANDLDLNFGGKNSSGNDIDFNKIKADAREKYL
chicken Type G	type IB	CP61	261	FNLMKGIDYYTNSVIYNTKGYDAKNTEFYNRIDPYMERLESLCTIGDKLNNDNAWLVNNALYYTGRMGKFREDPSISQRALERAMKEYPYLSYQYIEAANDLDLNFGGKNSSGNDIDFNKIKADAREKYL
chicken Type G	type IB	D2	261	FNLMKGIDYYTNSVIYNTKGYDAKNTEFYNRIDPYMERLESLCTIGDKLNNDNAWLVNNALYYTGRMGKFREDPSISQRALERAMKEYPYLSYQYIEAANDLDLNFGGKNSSGNDIDFNKIKADAREKYL
chicken Type G	type IB	D15	261	FNLMKGIDYYTNSVIYNTKGYDAKNTEFYNRIDPYMERLESLCTIGDKLNNDNAWLVNNALYYTGRMGKFREDPSISQRALERAMKEYPYLSYQYIEAANDLDLNFGGKNSSGNDIDFNKIKADAREKYL
chicken Type G	type II	CP23	1	--
chicken Type G	type II	DEL1	1	--
chicken Type G	type II	D8	1	--
chicken Type A	type III	D13	261	FNLMKGIDYYTNSVIYNTKGYDAKNTEFYNRIDPYMERLESLCTIGDKLNNDNAWLVNNALYYTGRMGKFREDPSISQRALERAMKEYPYLSYQYIEAANDLDLNFGGKNSSGNDIDFNKIKADAREKYL
chicken Type A	full length	CP24	261	FNLMKGIDYYTNSVIYNTKGYDAKNTEFYNRIDPYMERLESLCTIGDKLNNDNAWLVNNALYYTGRMGKFREDPSISQRALERAMKEYPYLSYQYIEAANDLDLNFGGKNSSGNDIDFNKIKADAREKYL
chicken Type A	full length	CP43	261	FNLMKGIDYYTNSVIYNTKGYDAKNTEFYNRIDPYMERLESLCTIGDKLNNDNAWLVNNALYYTGRMGKFREDPSISQRALERAMKEYPYLSYQYIEAANDLDLNFGGKNSSGNDIDFNKIKADAREKYL
chicken Type A	full length	JIR4857	261	FNLMKGIDYYTNSVIYNTKGYDAKNTEFYNRIDPYMERLESLCTIGDKLNNDNAWLVNNALYYTGRMGKFREDPSISQRALERAMKEYPYLSYQYIEAANDLDLNFGGKNSSGNDIDFNKIKADAREKYL
chicken Type A	full length	S2	261	FNLMKGIDYYTNSVIYNTKGYDAKNTEFYNRIDPYMERLESLCTIGDKLNNDNAWLVNNALYYTGRMGKFREDPSISQEALERAMKEYPYLSYQYIEAANDLDLNFGGKDSSGNDIDFNNIKADARGKYL
chicken Type A	full length	PK406	261	FNLMKGIDYYTNSVIYNTKGYDAKNTEFYNRIDPYMERLESLCTIGDKLNNDNAWLVNNALYYTGRMGKFREDPSISQRALERAMKEYPYLSYQYIEAANDLDLNFGGKNSSGNDIDFNKIKADAREKYL
chicken Type A	full length	JIR4874	261	FNLMKGIDYYTNSVIYNTKGYDAKNTEFYNRIDPYMERLESLCTIGDKLNNDNAWLVNNALYYTGRMGKFREDPSISQRALERAMKEYPYLSYQYIEAANDLDLNFGGKNSSGNDIDFNKIKADAREKYL
chicken Type A	type II	JGS4104	1	--
chicken Type A	type II	D4	1	--
chicken Type A	full length	AN68	261	FNLMKGIDYYTNSVIYNTKGYDAKNTEFYNRIDPYMERLESLCTIGDKLNNDNAWLVNNALYYTGRMGKFREDPSISQEALERAMKEYPYLSYQYIEAANDLDLNFGGKDSSGNDIDFNKIKADAREKYL
chicken Type A	full length	N11	261	FNLMKGIDYYTNSVIYNTKGYDAKNTEFYNRIDPYMERLESLCTIGDKLNNDNAWLVNNALYYTGRMGKFREDPSISQRALERAMKEYPYLSYQYIEAANDLDLNFGGKNSSGNDIDFNKIKADAREKYL
chicken Type A	full length	CP15	261	FNLMKGIDYYTNSVIYNTKGYDAKNTEFYNRIDPYMERLESLCTIGDKLNNDNAWLVNNALYYTGRMGKFREDPSISQRALERAMKEYPYLSYQYIEAANDLDLNFGGKNSSGNDIDFNKIKADAREKYL
		CONSENSUS	261	....

activator

peptidase

Figure S1 (cont.)

Group	Type	Strain	Start	Sequence
non-chicken	full length	1207CPER	391	PKTYTFDDGKFVVKAGDKVTEEKIKRLYWASKEVKAQFMRVVQNDKALEEGNPDDILTVVIYNSPEEYKLNRIINGFSTDNGGIYIENIGTFFTYERTPEESIYTLEELFRHEFTHYLQGRYVVPGMWGQ
	full length	JGS1721	391	PKTYTFDDGKFVVKAGDKVTEEKIKRLYWASKEVKAQFMRVVQNDKALEEGNPDDILTVVIYNSPEEYKLNRIINGFSTDNGGIYIENIGTFFTYERTPEESIYTLEELFRHEFTHYLQGRYVVPGMWGQ
	full length	F4969	391	PKTYTFDDGKFVVKAGDKVTEEKIKRLYWASKEVKAQFMRVVQNDKALEEGNPDDILTVVIYNSPEEYKLNRIINGFSTDNGGIYIENIGTFFTYERTPEESIYTLEELFRHEFTHYLQGRYVVPGMWGQ
	full length	SM101	391	PKTYTFDDGKFVVKAGDKVTEEKIKRLYWASKEVKAQFMRVVQNDKALEEGNPDDILTVVIYNSPEEYKLNRIINGFSTDNGGIYIENIGTFFTYERTPEESIYTLEELFRHEFTHYLQGRYVVPGMWGQ
	full length	FORC003	391	PKTYTFDDGKFVVKAGDKVTEEKIKRLYWASKEVKAQFMRVVQNDKALEEGNPDDILTVVIYNSPEEYKLNRIINGFSTDNGGIYIENIGTFFTYERTPEESIYTLEELFRHEFTHYLQGRYVVPGMWGQ
	full length	NCTC8239	391	PKTYTFDDGKFVVKAGDKVTEEKIKRLYWASKEVKAQFMRVVQNDKALEEGNPDDILTVVIYNSPEEYKLNRIINGFSTDNGGIYIENIGTFFTYERTPEESIYTLEELFRHEFTHYLQGRYVVPGMWGQ
	full length	JGS1987	391	PKTYTFDDGKFVVKAGDKVTEEKIKRLYWASKEVKAQFMRVVQNDKALEEGNPDDILTVVIYNSPEEYKLNRIINGFSTDNGGIYIENIGTFFTYERTPEESIYTLEELFRHEFTHYLQGRYVVPGMWGQ
	full length	STRAIN13	391	PKTYTFDDGKFVVKAGDKVTEEKIKRLYWASKEVKAQFMRVVQNDKALEEGNPDDILTVVIYNSPEEYKLNRIINGFSTDNGGIYIENIGTFFTYERTPEESIYTLEELFRHEFTHYLQGRYVVPGMWGQ
	full length	MJR7757A	391	PKTYTFDDGKFVVKAGDKVTEEKIKRLYWASKEVKAQFMRVVQNDKALEEGNPDDVLTVVIYNSPEEYKLNRIINGFSTDNGGIYIENIGTFFTYERTPEESIYTLEELFRHEFTHYLQGRYVVPGMWGQ
	full length	ATCC1324	391	PKTYTFDDGKFVVKAGDKVTEEKIKRLYWASKEVKAQFMRVVQNDKALEEGNPDDILTVVIYNSPEEYKLNRIINGFSTDNGGIYIENIGTFFTYERTPEESIYTLEELFRHEFTHYLQGRYVVPGMWGQ
	full length	JFP983	391	PKTYTFDDGKFVVKAGDKVTEEKIKRLYWASKEVKAQFMRVVQNDKALEEGNPDDILTVVIYNSPEEYKLNRIINGFSTDNGGIYIENIGTFFTYERTPEESIYTLEELFRHEFTHYLQGRYVVPGMWGQ
	full length	JGS1495	391	PKTYTFDDGKFVVKAGDKVTEEKIKRLYWASKEVKAQFMRVVQNDKALEEGNPDDILTVVIYNSPEEYKLNRIINGFSTDNGGIYIENIGTFFTYERTPEESIYTLEELFRHEFTHYLQGRYVVPGMWGQ
	full length	JJC	391	PKTYTFDDGKFVVKAGDKVTEEKIKRLYWASKEVKAQFMRVVQNDKALEEGNPDDILTVVIYNSPEEYKLNRIINGFSTDNGGIYIENIGTFFTYERTPEESIYTLEELFRHEFTHYLQGRYVVPGMWGQ
	full length	JFP727	391	PKTYTFDDGKFVVKAGDKVTEEKIKRLYWASKEVKAQFMRVVQNDKALEEGNPDDILTVVIYNSPEEYKLNRIINGFSTDNGGIYIENIGTFFTYERTPEESIYTLEELFRHEFTHYLQGRYVVPGMWGQ
	full length	WAL14572	391	PKTYTFDDGKFVVKAGDKVTEEKIKRLYWASKEVKAQFMRVVQNDKALEEGNPDDILTVVIYNSPEEYKLNRIINGFSTDNGGIYIENIGTFFTYERTPEESIYTLEELFRHEFTHYLQGRYVVPGMWGQ
	full length	JP838	391	PKTYTFDDGKFVVKAGDKVTEEKIKRLYWASKEVKAQFMRVVQNDKALEEGNPDDILTVVIYNSPEEYKLNRIINGFSTDNGGIYIENIGTFFTYERTPEESIYTLEELFRHEFTHYLQGRYVVPGMWGQ
	full length	JFP923	391	PKTYTFDDGKFVVKAGDKVTEEKIKRLYWASKEVKAQFMRVVQNDKALEEGNPDDILTVVIYNSPEEYKLNRIINGFSTDNGGIYIENIGTFFTYERTPEESIYTLEELFRHEFTHYLQGRYVVPGMWGQ
	full length	NCTC13170	391	PKTYTFDDGKFVVKAGDKVTEEKIKRLYWASKEVKAQFMRVVQNDKALEEGNPDDILTVVIYNSPEEYKLNRIINGFSTDNGGIYIENIGTFFTYERTPEESIYTLEELFRHEFTHYLQGRYVVPGMWGQ
	full length	MGYG-HGUT-02372	391	PKTYTFDDGKFVVKAGDKVTEEKIKRLYWASKEVKAQFMRVVQNDKALEEGNPDDILTVVIYNSPEEYKLNRIINGFSTDNGGIYIENIGTFFTYERTPEESIYTLEELFRHEFTHYLQGRYVVPGMWGQ
	full length	JXJA17	391	PKTYTFDDGKFVVKAGDKVTEEKIKRLYWASKEVKAQFMRVVQNDKALEEGNPDDILTVVIYNSPEEYKLNRIINGFSTDNGGIYIENIGTFFTYERTPEESIYTLEELFRHEFTHYLQGRYVVPGMWGQ
	full length	4928STDY7387940	391	PKTYTFDDGKFVVKAGDKVTEEKIKRLYWASKEVKAQFMRVVQNDKALEEGNPDDILTVVIYNSPEEYKLNRIINGFSTDNGGIYIENIGTFFTYERTPEESIYTLEELFRHEFTHYLQGRYVVPGMWGQ
	full length	NCTC2837	391	PKTYTFDDGKFVVKAGDKVTEEKIKRLYWASKEVKAQFMRVVQNDKALEEGNPDDILTVVIYNSPEEYKLNRIINGFSTDNGGIYIENIGTFFTYERTPEESIYTLEELFRHEFTHYLQGRYVVPGMWGQ
	type IV	JP55	391	PKTYTFDDGKFVVKAGDKVTEEKIKRLYWASKEVKAQFMRVVQNDKALEEGNPDDILTVVIYNSPEEYKLNRIINGFSTDNGGIYIENIGTFFTYERTPEESIYTLEELFRHEFTHYLQGRYVVPGMWGQ
chicken Type G	full length	D3	391	PKTYTFDDGKFVVKAGDKVTEEKIKRLYWASKEVKAQFMRVVQNDKALEEGNPDDILTVVIYNSPEEYKLNRIINGFSTDNGGIYIENIGTFFTYERTPEESIYTLEELFRHEFTHYLQGRYVVPGMWGQ
	type IA	CP56	391	PKTYTFDDGKFVVKAGDKVTEEKIKRLYWASKEVKAQFMRVVQNDKALEEGNPDDILTVVIYNSPEEYKLNRIINGFSTDNGGIYIENIGTFFTYERTPEESIYTLEELFRHEFTHYLQGRYVVPGMWGQ
	type IA	JIR4860	391	PKTYTFDDGKFVVKAGDKVTEEKIKRLYWASKEVKAQFMRVVQNDKALEEGNPDDILTVVIYNSPEEYKLNRIINGFSTDNGGIYIENIGTFFTYERTPEESIYTLEELFRHEFTHYLQGRYVVPGMWGQ
	type IA	JIR4869	391	PKTYTFDDGKFVVKAGDKVTEEKIKRLYWASKEVKAQFMRVVQNDKALEEGNPDDILTVVIYNSPEEYKLNRIINGFSTDNGGIYIENIGTFFTYERTPEESIYTLEELFRHEFTHYLQGRYVVPGMWGQ
	type IA	JIRE4866	391	PKTYTFDDGKFVVKAGDKVTEEKIKRLYWASKEVKAQFMRVVQNDKALEEGNPDDILTVVIYNSPEEYKLNRIINGFSTDNGGIYIENIGTFFTYERTPEESIYTLEELFRHEFTHYLQGRYVVPGMWGQ
	type IA	PM69	391	PKTYTFDDGKFVVKAGDKVTEEKIKRLYWASKEVKAQFMRVVQNDKALEEGNPDDILTVVIYNSPEEYKLNRIINGFSTDNGGIYIENIGTFFTYERTPEESIYTLEELFRHEFTHYLQGRYVVPGMWGQ
	type IB	CP60	391	PKTYTFDDGKFVVKAGDKVTEEKIKRLYWASKEVKAQFMRVVQNDKALEEGNPDDILTVVIYNSPEEYKLNRIINGFSTDNGGIYIENIGTFFTYERTPEESIYTLEELFRHEFTHYLQGRYVVPGMWGQ
	type IB	CP61	391	PKTYTFDDGKFVVKAGDKVTEEKIKRLYWASKEVKAQFMRVVQNDKALEEGNPDDILTVVIYNSPEEYKLNRIINGFSTDNGGIYIENIGTFFTYERTPEESIYTLEELFRHEFTHYLQGRYVVPGMWGQ
	type IB	D2	391	PKTYTFDDGKFVVKAGDKVTEEKIKRLYWASKEVKAQFMRVVQNDKALEEGNPDDILTVVIYNSPEEYKLNRIINGFSTDNGGIYIENIGTFFTYERTPEESIYTLEELFRHEFTHYLQGRYVVPGMWGQ
	type IB	D15	391	PKTYTFDDGKFVVKAGDKVTEEKIKRLYWASKEVKAQFMRVVQNDKALEEGNPDDILTVVIYNSPEEYKLNRIINGFSTDNGGIYIENIGTFFTYERTPEESIYTLEELFRHEFTHYLQGRYVVPGMWGQ
	type II	CP23	1	---MKEHQRKVYTLEELFRHEFTHYLQGRYVVPGMWGQ
	type II	DEL1	1	---MKEHQRKVYTLEELFRHEFTHYLQGRYVVPGMWGQ
	type II	D8	1	---MKEHQRKVYTLEELFRHEFTHYLQGRYVVPGMWGQ
	type III	D13	391	PKTYTFDDGKFVVKAGDKVTEEKIKRLYWASKEVKAQFMRVVQNDKALEEGNPDDILTVVIYNSPEEYKLNRIINGFSTDNGGIYIENIGTFFTYERTPEESIYTLEELFRHEFTHYLQGRYVVPGMWGQ
chicken Type A	full length	CP24	391	PKTYTFDDGKFVVKAGDKVTEEKIKRLYWASKEVKAQFMRVVQNDKALEEGNPDDILTVVIYNSPEEYKLNRIINGFSTDNGGIYIENIGTFFTYERTPEESIYTLEELFRHEFTHYLQGRYVVPGMWGQ
	full length	CP43	391	PKTYTFDDGKFVVKAGDKVTEEKIKRLYWASKEVKAQFMRVVQNDKALEEGNPDDILTVVIYNSPEEYKLNRIINGFSTDNGGIYIENIGTFFTYERTPEESIYTLEELFRHEFTHYLQGRYVVPGMWGQ
	full length	JIR4857	391	PKTYTFDDGKFVVKAGDKVTEEKIKRLYWASKEVKAQFMRVVQNDKALEEGNPDDILTVVIYNSPEEYKLNRIINGFSTDNGGIYIENIGTFFTYERTPEESIYTLEELFRHEFTHYLQGRYVVPGMWGQ
	full length	S2	391	PKTYTFDDGKFVVKAGDKVTEEKIKRLYWASKEVKAQFMRVVQNDKALEEGNPDDILTVVIYNSPEEYKLNRIINGFSTDNGGIYIENIGTFFTYERTPEESIYTLEELFRHEFTHYLQGRYVVPGMWGQ
	full length	PK406	391	PKTYTFDDGKFVVKAGDKVTEEKIKRLYWASKEVKAQFMRVVQNDKALEEGNPDDILTVVIYNSPEEYKLNRIINGFSTDNGGIYIENIGTFFTYERTPEESIYTLEELFRHEFTHYLQGRYVVPGMWGQ
	full length	JIR4874	391	PKTYTFDDGKFVVKAGDKVTEEKIKRLYWASKEVKAQFMRVVQNDKALEEGNPDDILTVVIYNSPEEYKLNRIINGFSTDNGGIYIENIGTFFTYERTPEESIYTLEELFRHEFTHYLQGRYVVPGMWGQ
	type II	JGS4104	1	---MKEHQRKVYTLEELFRHEFTHYLQGRYVVPGMWGQ
	type II	D4	1	---MKEHQRKVYTLEELFRHEFTHYLQGRYVVPGMWGQ
	full length	AN68	391	PKTYTFDDGKFVVKAGDKVTEEKIKRLYWASKEVKAQFMRVVQNDKALEEGNPDDILTVVIYNSPEEYKLNRIINGFSTDNGGIYIENIGTFFTYERTPEESIYTLEELFRHEFTHYLQGRYVVPGMWGQ
	full length	N11	391	PKTYTFDDGKFVVKAGDKVTEEKIKRLYWASKEVKAQFMRVVQNDKALEEGNPDDILTVVIYNSPEEYKLNRIINGFSTDNGGIYIENIGTFFTYERTPEESIYTLEELFRHEFTHYLQGRYVVPGMWGQ
	full length	CP15	391	PKTYTFDDGKFVVKAGDKVTEEKIKRLYWASKEVKAQFMRVVQNDKALEEGNPDDILTVVIYNSPEEYKLNRIINGFSTDNGGIYIENIGTFFTYERTPEESIYTLEELFRHEFTHYLQGRYVVPGMWGQ
		CONSENSUS	391	....***************************

peptidase

double glycine motif

HEXXH motif

Figure S1 (cont.)

Group	Type	Strain	Position	Sequence
non-chicken	full length	1207CPER	521	GEFYQEGVLTWYEEGTAEFFAGSTRTDGIKPRKSVTQGLAYDRNNRMSLYGVLHAKYGSWDFYNYGFALSNYMYNNNMGMFNKMTNYIKNNDVSGYKDYIASMSSDYGLNDRYQDYMDSLLNNIENLDVP
non-chicken	full length	JGS1721	521	GEFYQEGVLTWYEEGTAEFFAGSTRTDGIKPRKSVTQGLAYDRNNRMSLYGVLHAKYGSWDFYNYGFALSNYMYNNNMGMFNKMTNYIKNNDVSGYKDYIASMSSDYGLNDKYQDYMDSLLNNIDNLDVP
non-chicken	full length	F4969	521	GEFYQEGVLTWYEEGTAEFFAGSTRTDGIKPRKSVTQGLAYDRNNRMSLYGVLHAKYGSWDFYNYGFALSNYMYNNNMGMFNKMTNYIKNNDVSGYKDYIASMSSDYGLNDKYQDYMDSLLNNIDNLDVP
non-chicken	full length	SM101	521	GEFYQEGVLTWYEEGTAEFFAGSTRTDGIKPRKSVTQGLAYDRNNRMSLYDVLHAKYGSWDFYNYGFALSNYMYNNNIGMFNKMTNYIKNNDVSGYKDYIASMSSDYGLNDKYQDYIDSLLNNIDNLDVP
non-chicken	full length	FORC003	521	GEFYQEGVLTWYEEGTAEFFAGSTRTDGIKPRKSVTQGLAYDRNNRMSLYGVLHAKYGSWDFYNYGFALSNYMYNNNMGMFNKMTNYIKNNDVSGYKDYIASMSSDYGLNDRYQDYMDSLLNNIDNLDVP
non-chicken	full length	NCTC8239	521	GEFYQEGVLTWYEEGTAEFFAGSTRTDGIKPRKSVTQGLAYDRNNRMSLYGVLHAKYGSWDFYNYGFALSNYMYNNNMGMFNKMTNYIKNNDVSGYKDYIASMSSDYGLNDKYQDYMDSLLNNIDNLDVP
non-chicken	full length	JGS1987	521	GEFYQEGVLTWYEEGTAEFFAGSTRTDGIKPRKSVTQGLAYDRNNRMSLYGVLHAKYGSWDFYNYGFALSNYMYNNNMGMFNKMTNYIKNNDVSGYKDYIASMSSDYGLNDKYQDYMDSLLNNIDNLDVP
non-chicken	full length	STRAIN13	521	GEFYQEGVLTWYEEGTAEFFAGSTRTDGIKPRKSVTQGLAYDRNNRMSLYGVLHAKYGSWDFYNYGFALSNYMYNNNMGMFNKMTNYIKNNDVSGYKDYIASMSSDYGLNDKYQDYMDSLLNNIDNLDVP
non-chicken	full length	MJR7757A	521	GEFYQEGVLTWYEEGTAEFFAGSTRTDGIKPRKSVTQGLAYDRNNRMSLYGVLHAKYGSWDFYNYGFALSNYMYNNNMGMFNKMTNYIKNNDISGYKDYIASMSSDYGLNDRYQDYMDSLINNIDNLDVP
non-chicken	full length	ATCC1324	521	GEFYQEGVLTWYEEGTAEFFAGSTRTDGIKPRKSVTQGLAYDRNNRMSLYDVLHAKYGSWDFYNYGFALSNYMYNNNIGMFNKMTNYIKNNDVSGYKDYIASMSSDYGLNDKYQDYIDSLLNNIDNLDVP
non-chicken	full length	JFP983	521	GEFYQEGVLTWYEEGTAEFFAGSTRTDGIKPRKSVTQGLAYDRNNRMSLYGVLHAKYGSWDFYNYGFALSNYMYNNNMGMFNKMTNYIKNNDVSGYKDYIASMSSDYGLNDRYQDYMDSLLNNIDNLDVP
non-chicken	full length	JGS1495	521	GEFYQDGVLTWYEEGTAEFFAGSTRTDGIKPRKSVTQGLAYDRNNRMSLYDVLHAKYGSWDFYNYGFALSNYMYNNNIGMFNKMTNYIKNNDVSGYKDYIASMSSDYGLNDKYQDYIDSLLNNIDNLDVP
non-chicken	full length	JJC	521	GEFYQEGVLTWYEEGTAEFFAGSTRTDGIKPRKSVTQGLAYDRNNRMSLYGVLHAKYGSWDFYNYGFALSNYMYNNNMGMFNKMTNYIKNNDVSGYKDYIASMSSDYGLNDKYQDYMDSLLNNIDNLDVP
non-chicken	full length	JFP727	521	GEFYQEGVLTWYEEGTAEFFAGSTRTDGIKPRKSVTQGLAYDRNNRMSLYGVLHAKYGSWDFYNYGFALSNYMYNNNMGMFNKMTNYIKNNDVSGYKDYIASMSSDYGLNDRYQDYMDSLLNNIDNLDVP
non-chicken	full length	WAL14572	521	GEFYQEGVLTWYEEGTAEFFAGSTRTDGIKPRKSVTQGLAYDRNNRMSLYGVLHAKYGSWDFYNYGFALSNYMYNNNMGMFNKMTNYIKNNDVSGYKDYIASMSSDYGLNDKYQDYMDSLLNNIDNLDVP
non-chicken	full length	JP838	521	GEFYQEGVLTWYEEGTAEFFAGSTRTDGIKPRKSVTQGLAYDRNNRMSLYGVLHAKYGSWDFYNYGFALSNYMYNNNMGMFNKMTNYIKNNDVSGYKDYIASMSSDYGLNDKYQDYMDSLLNNIDNLDVP
non-chicken	full length	JFP923	521	GEFYQEGVLTWYEEGTAEFFAGSTRTDGIKPRKSVTQGLAYDRNNRMSLYGVLHAKYGSWDFYNYGFALSNYMYNNNMGMFNKMTNYIKNNDVSGYKDYIASMSSDYGLNDRYQDYMDSLLNNIDNLDVP
non-chicken	full length	NCTC13170	521	GEFYQEGVLTWYEEGTAEFFAGSTRTDGIKPRKSVTQGLAYDRNNRMSLYGVLHAKYGSWDFYNYGFALSNYMYNNNMGMFNKMTNYIKNNDVSGYKDYIASMSSDYGLNDRYQDYMDSLLNNIENLDVP
non-chicken	full length	MGYG-HGUT-02372	521	GEFYQEGVLTWYEEGTAEFFAGSTRTDGIKPRKSVTQGLAYDRNNRMSLYGVLHAKYGSWDFYNYGFALSNYMYNNNMGMFNKMTNYIKNNDVSGYKDYIASMSSDYGLNDKYQDYMDSLLNNIDNLDVP
non-chicken	full length	JXJA17	521	GEFYQDGVLTWYEEGTAEFFAGSTRTDGIKPRKSVTQGLAYDRNNRMSLYDVLHAKYGSWDFYNYGFALSNYMYNNNIGMFNKMTNYIKNNDVSGYKDYIASMSSDYGLNDKYQDYIDSLLNNIDNLDVP
non-chicken	full length	4928STDY7387940	521	GEFYQEGVLTWYEEGTAEFFAGSTRTDGIKPRKSVTQGLAYDRNNRMSLYDVLHAKYGSWDFYNYGFALSNYMYNNNIGMFNKMTNYIKNNDVSGYKDYIASMSSDYGLNDKYQDYIDSLLNNIDNLDVP
non-chicken	full length	NCTC2837	521	GEFYQEGVLTWYEEGTAEFFAGSTRTDGIKPRKSVTQGLAYDRNNRMSLYGVLHAKYGSWDFYNYGFALSNYMYNNNMGMFNKMTNYIKNNDVSGYKDYIASMSSDYGLNDKYQDYMDSLLNNIDNLDVP
non-chicken	type IV	JP55	521	GEFYQEGVLTWYEEGTAEFFAGSTRTDGIKPRKSVTQGLAYDRNNRMSLYGVLHAKYGSWDFYNYGFALSNYMYNNNMGMFNKMTNYIKNNDVSGYKDYIASMSSDYGLNDRYQDYMDSLLNNIENLDVP
chicken Type G	full length	D3	521	GEFYQEGVLTWYEEGTAEFFAGSTRTDGIKPRKSVTQGLAYDRNNRMSLYGVLHAKYGSWDFYNYGFALSNYMYNNNMGMFNKMTNYIKNNDVSGYKDYIASMSSDYGLNDRYQDYMDSLLNNIENLDVP
chicken Type G	type IA	CP56	521	GEFYQEGVLTWYEEGTAEFFAGSTRTDGIKPRKSVTQGLAYDRNNRMSLYGVLHAKYGSWDFYNYGFALSNYMYNNNMGMFNKMTNYIKNNDVSGYKDYIASMSSDYGLNDRYQDYMDSLLNNIENLDVP
chicken Type G	type IA	JIR4860	521	GEFYQEGVLTWYEEGTAEFFAGSTRTDGIKPRKSVTQGLAYDRNNRMSLYGVLHAKYGSWDFYNYGFALSNYMYNNNMGMFNKMTNYIKNNDVSGYKDYIASMSSDYGLNDRYQDYMDSLLNNIENLDVP
chicken Type G	type IA	JIR4869	521	GEFYQEGVLTWYEEGTAEFFAGSTRTDGIKPRKSVTQGLAYDRNNRMSLYGVLHAKYGSWDFYNYGFALSNYMYNNNMGMFNKMTNYIKNNDVSGYKDYIASMSSDYGLNDRYQDYMDSLLNNIENLDVP
chicken Type G	type IA	JIRE4866	521	GEFYQEGVLTWYEEGTAEFFAGSTRTDGIKPRKSVTQGLAYDRNNRMSLYGVLHAKYGSWDFYNYGFALSNYMYNNNMGMFNKMTNYIKNNDVSGYKDYIASMSSDYGLNDRYQDYMDSLLNNIENLDVP
chicken Type G	type IA	PM69	521	GEFYQEGVLTWYEEGTAEFFAGSTRTDGIKPRKSVTQGLAYDRNNRMSLYGVLHAKYGSWDFYNYGFALSNYMYNNNMGMFNKMTNYIKNNDVSGYKDYIASMSSDYGLNDRYQDYMDSLLNNIENLDVP
chicken Type G	type IB	CP60	521	GEFYQEGVLTWYEEGTAEFFAGSTRTDGIKPRKSVTQGLAYDRNNRMSLYGVLHAKYGSWDFYNYGFALSNYMYNNNMGMFNKMTNYIKNNDVSGYKDYIASMSSDYGLNDKYQDYMDSLLNNIDNLDVP
chicken Type G	type IB	CP61	521	GEFYQEGVLTWYEEGTAEFFAGSTRTDGIKPRKSVTQGLAYDRNNRMSLYGVLHAKYGSWDFYNYGFALSNYMYNNNMGMFNKMTNYIKNNDVSGYKDYIASMSSDYGLNDKYQDYMDSLLNNIDNLDVP
chicken Type G	type IB	D2	521	GEFYQEGVLTWYEEGTAEFFAGSTRTDGIKPRKSVTQGLAYDRNNRMSLYGVLHAKYGSWDFYNYGFALSNYMYNNNMGMFNKMTNYIKNNDVSGYKDYIASMSSDYGLNDKYQDYMDSLLNNIDNLDVP
chicken Type G	type IB	D15	521	GEFYQEGVLTWYEEGTAEFFAGSTRTDGIKPRKSVTQGLAYDRNNRMSLYGVLHAKYGSWDFYNYGFALSNYMYNNNMGMFNKMTNYIKNNDVSGYKDYIASMSSDYGLNDKYQDYMDSLLNNIDNLDVP
chicken Type G	type II	CP23	36	GEFYQEGVLTWYEEGTAEFFAGSTRTDGIKPRKSVTQGLAYDRNNRMSLYGVLHAKYGSWDFYNYGFALSNYMYNNNMGMFNKMTNYIKNNDVSSYKDYIASMSSDYGLNDKYQDYMDSLLNNIDNLDVP
chicken Type G	type II	DEL1	36	GEFYQEGVLTWYEEGTAEFFAGSTRTDGIKPRKSVTQGLAYDRNNRMSLYGVLHAKYGSWDFYNYGFALSNYMYNNNMGMFNKMTNYIKNNDVSSYKDYIASMSSDYGLNDKYQDYMDSLLNNIDNLDVP
chicken Type G	type II	D8	36	GEFYQEGVLTWYEEGTAEFFAGSTRTDGIKPRKSVTQGLAYDRNNRMSLYGVLHAKYGSWDFYNYGFALSNYMYNNNMGMFNKMTNYIKNNDVSSYKDYIASMSSDYGLNDKYQDYMDSLLNNIDNLDVP
chicken Type G	type III	D13	521	GEFYQEGVLTWYEEGTAEFFAGSTRTDGIKPRKSVTQGLAYDRNNRMSLYGVLHAKYGSWDFYNYGFALSNYMYNNNMGMFNKMTNYIKNNDVSGYKDYIASMSSDYGLNDKYQDYMDSLLNNIDNLDVP
chicken Type A	full length	CP24	521	GEFYQEGVLTWYEEGTAEFFAGSTRTDGIKPRKSVTQGLAYDRNNRMSLYGVLHAKYGSWDFYNYGFALSNYMYNNNMGMFNKMTNYIKNNDVSGYKDYIASMSSDYGLNDRYQDYMDSLLNNIENLDVP
chicken Type A	full length	CP43	521	GEFYQEGVLTWYEEGTAEFFAGSTRTDGIKPRKSVTQGLAYDRNNRMSLYGVLHAKYGSWDFYNYGFALSNYMYNNNMGMFNKMTNYIKNNDVSGYKDYIASMSSDYGLNDKYQDYMDSLLNNIDNLDVP
chicken Type A	full length	JIR4857	521	GEFYQEGVLTWYEEGTAEFFAGSTRTDGIKPRKSVTQGLAYDRNNRMSLYGVLHAKYGSWDFYNYGFALSNYMYNNNMGMFNKMTNYIKNNDVSGYKDYIASMSSDYGLNDKYQDYMDSLLNNIDNLDVP
chicken Type A	full length	S2	521	GEFYQEGVLTWYEEGTAEFFAGSTRTDGIKPRKSVTQGLAYDRNNRMSLYGVLHAKYGSWDFYNYGFAISNYMYNNNMRMFNKMTNYIKNNDVSGYKDYIASMSSDYGLNDRYQDYMDSLLNNIDNLDVP
chicken Type A	full length	PK406	521	GEFYQEGVLTWYEEGTAEFFAGSTRTDGIKPRKSVTQGLAYDRNNRMSLYGVLHAKYGSWDFYNYGFALSNYMYNNNMGMFNKMTNYIKNNDVSGYKDYIASMSSDYGLNDRYQDYMDSLLNNIENLDVP
chicken Type A	full length	JIR4874	521	GEFYQEGVLTWYEEGTAEFFAGSTRTDGIKPRKSVTQGLAYDRNNRMSLYGVLHAKYGSWDFYNYGFALSNYMYNNNMGMFNKMTNYIKNNDVSGYKDYIASMSSDYGLNDRYQDYMDSLLNNIENLDVP
chicken Type A	type II	JGS4104	36	GEFYQEGVLTWYEEGTAEFFAGSTRTDGIKPRKSVTQGLAYDRNNRMSLYGVLHAKYGSWDFYNYGFALSNYMYNNNMGMFNKMTNYIKNNDVSSYKDYIASMSSDYGLNDKYQDYMDSLLNNIDNLDVP
chicken Type A	type II	D4	36	GEFYQEGVLTWYEEGTAEFFAGSTRTDGIKPRKSVTQGLAYDRNNRMSLYGVLHAKYGSWDFYNYGFALSNYMYNNNMGMFNKMTNYIKNNDVSSYKDYIASMSSDYGLNDKYQDYMDSLLNNIDNLDVP
chicken Type A	full length	AN68	521	GEFYQEGVLTWYEEGTAEFFAGSTRTDGIKPRKSVTQGLAYDRNNRMSLYGVLHAKYGSWDFYNYGFALSNYMYNNNMGMFNKMTNYIKNNDVSGYKDYIASMSSDYGLNDRYQDYMDSLLNNIDNLDVP
chicken Type A	full length	N11	521	GEFYQEGVLTWYEEGTAEFFAGSTRTDGIKPRKSVTQGLAYDRNNRMSLYGVLHAKYGSWDFYNYGFALSNYMYNNNMGMFNKMTNYIKNNDVSGYKDYIASMSSDYGLNDRYQDYMDSLLNNIENLDVP
chicken Type A	full length	CP15	521	GEFYQEGVLTWYEEGTAEFFAGSTRTDGIKPRKSVTQGLAYDRNNRMSLYGVLHAKYGSWDFYNYGFALSNYMYNNNMGMFNKMTNYIKNNDVSGYKDYIASMSSDYGLNDRYQDYMDSLLNNIENLDVP
		CONSENSUS	521	[illegible]

third zinc ligand motif

peptidase

Figure S1 (cont.)

Group	Type	Strain	Start	Sequence
non-chicken	full length	1207CPER	651	LVSDEYVNGHEAKDINEITKDIKEVSNIKDLSSNVEKSQFFTTYDMRGTYVGGRSQGEENDWKDMNSKLNDILKELSKKSWNGYKTVTAYFVNHKVDENGNYVYDVVFHGMNTDTNTDVHVNKEPKAVIK
non-chicken	full length	JGS1721	651	LVSDEYVNGHEAKDINEITNDIKEVSNIKDLSSNVEKSQFFTTYDMRGTYVGGRSQGEENDWKDMNSKLNDILKELSKKSWNGYKTVTAYFVNHKVDGNGNYVYDVVFHGMDTDTNTDVHVNKEPKAVIK
non-chicken	full length	F4969	651	LVSDEYVNGHEAKDINEITKDIKEVSNIKDLSSNVEKSQFFTTYDMRGTYVGGRSQGEENDWKDMNSKLNYILKELSKKSWNGYKTVTAYFVNHKVDGNGNYVYDVVFHGMNTDTNTDVHVNKEPKAVIK
non-chicken	full length	SM101	651	SVSDEYVNGHEAKDINEITKDIKEVSNIKDLSSNVEKSQFFTTYDMRGTYVGGRSQGEENDWKDMNSKLNDILKELSKKSWNGYKTVTAYFVNHKVDENGNYVYDVVFHGMNTDTNTDVHVNKEPKAVIK
non-chicken	full length	FORC003	651	LVSDEYVNGHEAKDINEITNDIKEVSNIKDLSSNVEKSQFFTTYDMRGTYVGGRSQGEENDWKDMNSKLNDILKELSKKSWNGYKTVTAYFVNHKVDGNGNYVYDVVFHGMDTDTNTDVHVNKEPKAVIK
non-chicken	full length	NCTC8239	651	LVSDEYVNGHEAKDINEITKDIKEVSNIKDLSSNVEKSQFFTTYDMRGTYVGGRSQGEENDWKDMNSKLNDILKELSKKSWNGYKTVTAYFVNHKVDGNGNYVYDVVFHGMNTDTNTDVHVNKEPKAVIK
non-chicken	full length	JGS1987	651	LVSDEYVNGHEAKDINEITKDIKEVSNIKDLSSNVEKSQFFTTYDMKGTYVGGRSQGEENDWKDMNSKLNDILKELSKKSWNGYKTVTAYFVNHKVDENGNYVYDVVFHGMNTDTNTDVHVNKEPKAVIK
non-chicken	full length	STRAIN13	651	LVSDEYVNGHEAKDINEITNDIKEVSNIKDLSSNVEKSQFFTTYDMRGTYVGGRSQGEENDWKDMNSKLNDILKELSKKSWNGYKTVTAYFVNHKVDGNGNYVYDVVFHGMNTDTNTDVHVNKEPKAVIK
non-chicken	full length	MJR7757A	651	LVSDEYVNGHEAKDINEITKDIKEVSNINDLSSNVEKSQFFTTYDMRGTYIGVRSQGEENDWKDMNSKLNDILKELSKKSWNGYKTVTAYFVNHKVDENGNYVYDVVFHGMNTDTNTDVHVNKEPKAVIK
non-chicken	full length	ATCC1324	651	LVSDEYVNGHEAKDINEITNDIKEVSNIKDLSSNVEKSQFFTTYDMRGTYVGERSQGEENDWKDMNSKLNDILKELSKKSWNGYKTVTAYFVNHKVDENGNYVYDVVFHGMNTDTNTDVHVNKEPKAVIK
non-chicken	full length	JFP983	651	LVSDEYVNGHEAKDINEITKDIKEVSNIKDLSSNVEKSQFFTTYDMRGTYVGGRSQGEENDWKDMNSKLNDILKELSKKSWNGYKTVTAYFVNHKVDENGNYVYDVVFHGMNTDTNTDVHVNKEPKAVIK
non-chicken	full length	JGS1495	651	LVSDEYVNGHEAKDINEITNDIKEVSNIKDLSSNVEKSQFFTTYDMRGTYVGGRSQGEENDWKDMNSKLNDILKELSKKSWNGYKTVTAYFVNHKVDENGNYVYDVVFHGMNTDTNTDVHVNKEPKAVIK
non-chicken	full length	JJC	651	LVSDEYVNGHEAKDINEITKDIKEVSNIKDLSSNVEKSQFFTTYDMRGTYVGGRSQGEENDWKDMNSKLNDILKELSKKSWNGYKTVTAYFVNHKVDGNGNYVYDVVFHGMNTDTNTDVHVNKEPKAVIK
non-chicken	full length	JFP727	651	LVSDEYVNGHEAKDINEITKDIKEVSNIKDLSSNVEKSQFFTTYDMRGTYVGGRSQGEENDWKDMNSKLNDILKELSKKSWNGYKTVTAYFVNHKVDENGNYVYDVVFHGMNTDTNTDVHVNKEPKAVIK
non-chicken	full length	WAL14572	651	LVSDEYVNGHEAKDINEITNDIKEVSNIKDLSSNVEKSQFFTTYDMRGTYVGGRSQGEENDWKDMNSKLNDILKELSKKSWNGYKTVTAYFVNHKVDENGNYVYDVVFHGMNTDTNTDVHVNKEPKAVIK
non-chicken	full length	JP838	651	LVSDEYVNGHEAKDINEITKDIKEVSNIKDLSSNVEKSQFFTTYDMRGTYVGGRSQGEENDWKDMNSKLNDILKELSKKSWNGYKTVTAYFVNHKVDENGNYVYDVVFHGMNTDTNTDVHVNKEPKAVIK
non-chicken	full length	JFP923	651	LVSDEYVNGHEAKDINEITKDIKEVSNIKDLSSNVEKSQFFTTYDMRGTYVGGRSQGEENDWKDMNSKLNDILKELSKKSWNGYKTVTAYFVNHKVDENGNYVYDVVFHGMNTDTNTDVHVNKEPKAVIK
non-chicken	full length	NCTC13170	651	LVSDEYVNGHEAKDINEITKDIKEVSNIKDLSSNVEKSQFFTTYDMRGTYVGGRSQGEENDWKDMNSKLNDILKELSKKSWNGYKTVTAYFVNHKVDENGNYVYDVVFHGMNTDTNTDVHVNKEPKAVIK
non-chicken	full length	MGYG-HGUT-02372	651	LVSDEYVNGHEAKDINEITNDIKEVSNIKDLSSNVEKSQFFTTYDMRGTYVGGRSQGEENDWKDMNSKLNDILKELSKKSWNGYKTVTAYFVNHKVDENGNYVYDVVFHGMNTDTNTDVHVNKEPKAVIK
non-chicken	full length	JXJA17	651	LVSDEYVNGHEAKDINEITNDIKEVSNIKDLSSNVEKSQFFTTYDMRGTYVGGRSQGEENDWKDMNSKLNDILKELSKKSWNGYKTVTAYFVNHKVDENGNYVYDVVFHGMNTDTNTDVHVNKEPKAVIK
non-chicken	full length	4928STDY7387940	651	LVSDEYVNGHEAKDINEITNDIKEVSNIKDLSSNVEKSQFFTTYDMRGTYVGERSQGEENDWKDMNSKLNDILKELSKKSWNGYKTVTAYFVNHKVDENGNYVYDVVFHGMNTDTNTDVHVNKEPKAVIK
non-chicken	full length	NCTC2837	651	LVSDEYVNGHEAKDINEITNDIKEVSNIKDLSSNVEKSQFFTTYDMIGTYVGGRSQGEENDWKDMNSKLNDILKELSKKSWNGYKTVTAYFVNHKVDGNGNYVYDVVFHGMNTDTNTDVHVNKEPKAVIK
non-chicken	type IV	JP55	651	LVSDEYVNGHEAKDINEITKDIKEVSNIKDLSSNVEKSQFFTTYDMRGTYVGGRK---
chicken Type G	full length	D3	651	LVSDEYVNGHEAKDINEITKDIKEVSNIKDLSSNVEKYQFFTTYDMRGTYVGGRSQGEENDWKDMNSKLNDILKELSKKSWNGYKTVTAYFVNHKVDGNGNYVYDVVFHGMNTDTNTDVHVNKEPKAVIK
chicken Type G	type IA	CP56	651	LVSDEYVNGHEAKDINEITKDIKEVSNIKDLSSNVEKSQFFTTYDMRGTYVGGRSQGEENDWKDMNSKLNDILKELSKKSWNGYKTVTAYFVNHKVDENGNYVYDVVFHGMNTDTNTDVHVNKEPKAVIK
chicken Type G	type IA	JIR4860	651	LVSDEYVNGHEAKDINEITKDIKEVSNIKDLSSNVEKSQFFTTYDMRGTYVGGRSQGEENDWKDMNSKLNDILKELSKKSWNGYKTVTAYFVNHKVDENGNYVYDVVFHGMNTDTNTDVHVNKEPKAVIK
chicken Type G	type IA	JIR4869	651	LVSDEYVNGHEAKDINEITKDIKEVSNIKDLSSNVEKSQFFTTYDMRGTYVGGRSQGEENDWKDMNSKLNDILKELSKKSWNGYKTVTAYFVNHKVDENGNYVYDVVFHGMNTDTNTDVHVNKEPKAVIK
chicken Type G	type IA	JIRE4866	651	LVSDEYVNGHEAKDINEITKDIKEVSNIKDLSSNVEKSQFFTTYDMRGTYVGGRSQGEENDWKDMNSKLNDILKELSKKSWNGYKTVTAYFVNHKVDENGNYVYDVVFHGMNTDTNTDVHVNKEPKAVIK
chicken Type G	type IA	PM69	651	LVSDEYVNGHEAKDINEITKDIKEVSNIKDLSSNVEKSQFFTTYDMRGTYVGGRSQGEENDWKDMNSKLNDILKELSKKSWNGYKTVTAYFVNHKVDENGNYVYDVVFHGMNTDTNTDVHVNKEPKAVIK
chicken Type G	type IB	CP60	651	LVSDEYVNGHEAKDINEITKDIKEVSNIKDLSSNVEKSQFFTTYDMRGTYVGGRSQGEENDWKDMNSKLNYILKELSKKSWNGYKTVTAYFVNHKVDGNGNYVYDVVFHGMNTDTNTDVHVNKEPKAVIK
chicken Type G	type IB	CP61	651	LVSDEYVNGHEAKDINEITKDIKEVSNIKDLSSNVEKSQFFTTYDMRGTYVGGRSQGEENDWKDMNSKLNYILKELSKKSWNGYKTVTAYFVNHKVDGNGNYVYDVVFHGMNTDTNTDVHVNKEPKAVIK
chicken Type G	type IB	D2	651	LVSDEYVNGHEAKDINEITKDIKEVSNIKDLSSNVEKSQFFTTYDMRGTYVGGRSQGEENDWKDMNSKLNYILKELSKKSWNGYKTVTAYFVNHKVDGNGNYVYDVVFHGMNTDTNTDVHVNKEPKAVIK
chicken Type G	type IB	D15	651	LVSDEYVNGHEAKDINEITKDIKEVSNIKDLSSNVEKSQFFTTYDMRGTYVGGRSQGEENDWKDMNSKLNYILKELSKKSWNGYKTVTAYFVNHKVDGNGNYVYDVVFHGMNTDTNTDVHVNKEPKAVIK
chicken Type G	type II	CP23	166	LVSDEYVNGHEAKDINEITKDIKEVSNIKDLSSNVEKSQFFTTYDMRGTYVGGRSQGEENDWKDMNSKLNDILKELSKKSWNGYKTVTAYFVNHKVDGNGNYVYDVVFHGMNTDTNTDVHVNKEPKAVIK
chicken Type G	type II	DEL1	166	LVSDEYVNGHEAKDINEITKDIKEVSNIKDLSSNVEKSQFFTTYDMRGTYVGGRSQGEENDWKDMNSKLNDILKELSKKSWNGYKTVTAYFVNHKVDGNGNYVYDVVFHGMNTDTNTDVHVNKEPKAVIK
chicken Type G	type II	D8	166	LVSDEYVNGHEAKDINEITKDIKEVSNIKDLSSNVEKSQFFTTYDMRGTYVGGRSQGEENDWKDMNSKLNDILKELSKKSWNGYKTVTAYFVNHKVDGNGNYVYDVVFHGMNTDTNTDVHVNKEPKAVIK
chicken Type G	type III	D13	651	LVSDEYVNGHEAKDINEITKDIKEVSNIKDLSSNVEKSQFFTTYDMRGTYVGGRSQGEENDWKDMNSKLNDILKELSKKSWNGYKTVTAYFVNHKVDGNGNYVYDVVFHGMNTDTNTDVHVNKEPKAVIK
chicken Type A	full length	CP24	651	LVSDEYVNGHEAKDINEITKDIKEVSNIKDLSSNVEKSQFFTTYDMRGTYVGGRSQGEENDWKDMNSKLNDILKELSKKSWNGYKTVTAYFVNHKVDENGNYVYDVVFHGMNTDTNTDVHVNKEPKAVIK
chicken Type A	full length	CP43	651	LVSDEYVNGHEAKDINEITKDIKEVSNIKDLSSNVEKSQFFTTYDMRGTYVGGRSQGEENDWKDMNSKLNDILKELSKKSWNGYKTVTAYFVNHKVDENGNYVYDVVFHGMNTDTNTDVHVNKEPKAVIK
chicken Type A	full length	JIR4857	651	LVSDEYVNGHEAKDINEITKDIKEVSNIKDLSSNVEKSQFFTTYDMRGTYVGGRSQGEENDWKDMNSKLNYILKELSKKSWNGYKTVTAYFVNHKVDGNGNYVYDVVFHGMNTDTNTDVHVNKEPKAVIK
chicken Type A	full length	S2	651	LVSDEYVNGHEAKDINEITKDIKEVSNIKDLSSNVEKSQFFTTYDMRGTYVGGRSQGEENDWKDMNSKLNDILKELSKKSWNGYKTVTAYFVNHKVDENGNYVYDVVFHGMNTDTNTDVHVNKEPKAVIK
chicken Type A	full length	PK406	651	LVSDEYVNGHEAKDINEITKDIKEVSNIKDLSSNVEKSQFFTTYDMRGTYVGGRSQGEENDWKDMNSKLNDILKELSKKSWNGYKTVTAYFVNHKVDENGNYVYDVVFHGMNTDTNTDVHVNKEPKAVIK
chicken Type A	full length	JIR4874	651	LVSDEYVNGHEAKDINEITKDIKEVSNIKDLSSNVEKSQFFTTYDMRGTYVGGRSQGEENDWKDMNSKLNDILKELSKKSWNGYKTVTAYFVNHKVDENGNYVYDVVFHGMNTDTNTDVHVNKEPKAVIK
chicken Type A	type II	JGS4104	166	LVSDEYVNGHEAKDINEITKDIKEVSNIKDLSSNVEKSQFFTTYDMRGTYVGGRSQGEENDWKDMNSKLNDILKELSKKSWNGYKTVTAYFVNHKVDGNGNYVYDVVFHGMNTDTNTDVHVNKEPKAVIK
chicken Type A	type II	D4	166	LVSDEYVNGHEAKDINEITKDIKEVSNIKDLSSNVEKSQFFTTYDMRGTYVGGRSQGEENDWKDMNSKLNDILKELSKKSWNGYKTVTAYFVNHKVDGNGNYVYDVVFHGMNTDTNTDVHVNKEPKAVIK
chicken Type A	full length	AN68	651	LVSDEYVNGHEAKDINEITKDIKEVSNIKDLSSNVEKSQFFTTYDMRGTYVGGRSQGEENDWKDMNSKLNDILKELSKKSWNGYKTVTAYFVNHKVDGNGNYVYDVVFHGMNTDTNTDVHVNKEPKAVIK
chicken Type A	full length	N11	651	LVSDEYVNGHEAKDINEITKDIKEVSNIKDLSSNVEKSQFFTTYDMRGTYVGGRSQGEENDWKDMNSKLNDILKELSKKSWNGYKTVTAYFVNHKVDENGNYVYDVVFHGMNTDTNTDVHVNKEPKAVIK
chicken Type A	full length	CP15	651	LVSDEYVNGHEAKDINEITKDIKEVSNIKDLSSNVEKSQFFTTYDMRGTYVGGRSQGEENDWKDMNSKLNDILKELSKKSWNGYKTVTAYFVNHKVDGNGNYVYDVVFHGMNTDTNTDVHVNKEPKAVIK
		CONSENSUS	651	.******************.********.********.********.***.*.*..

helper

PKD domains

Figure S1 (cont.)

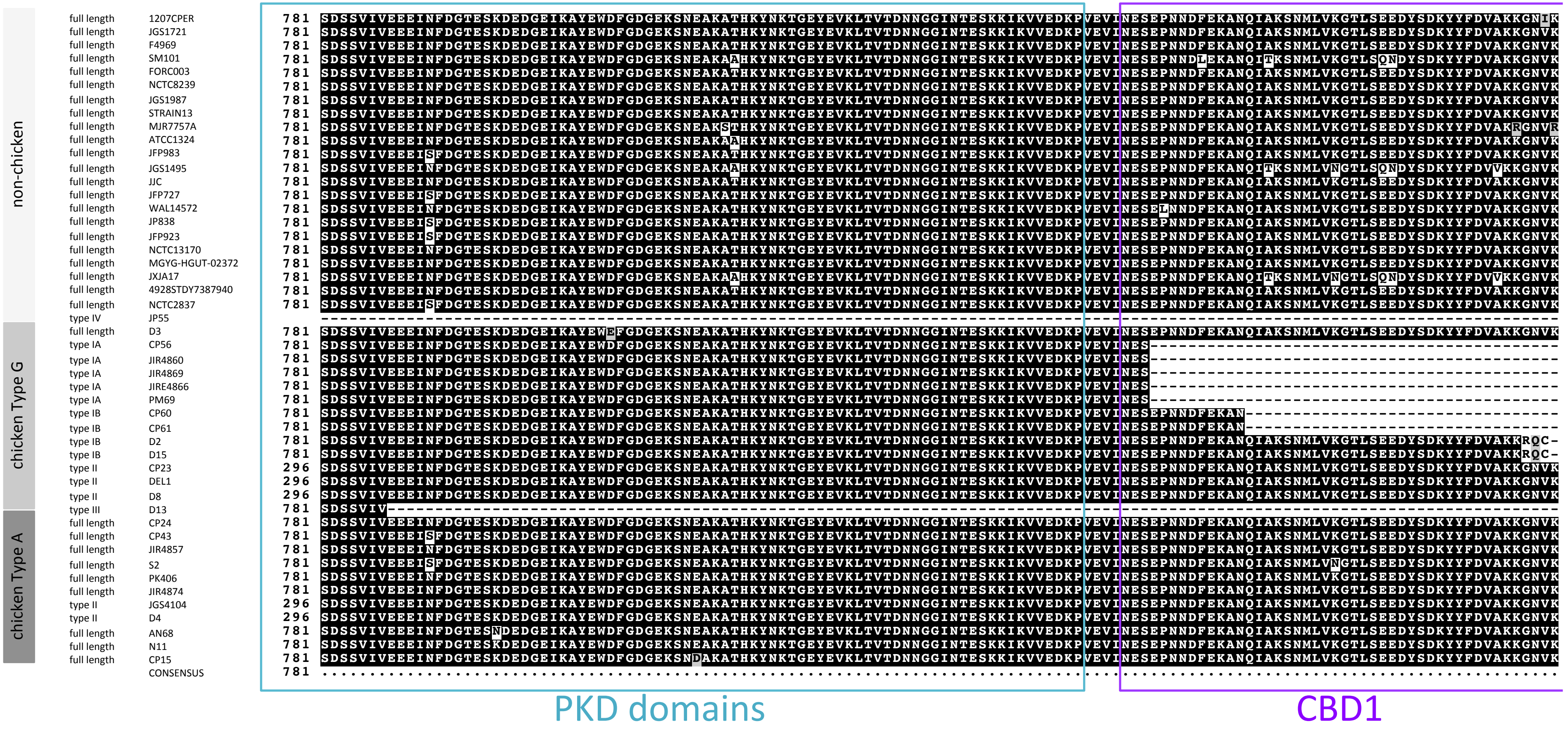

Figure S1 (cont.)

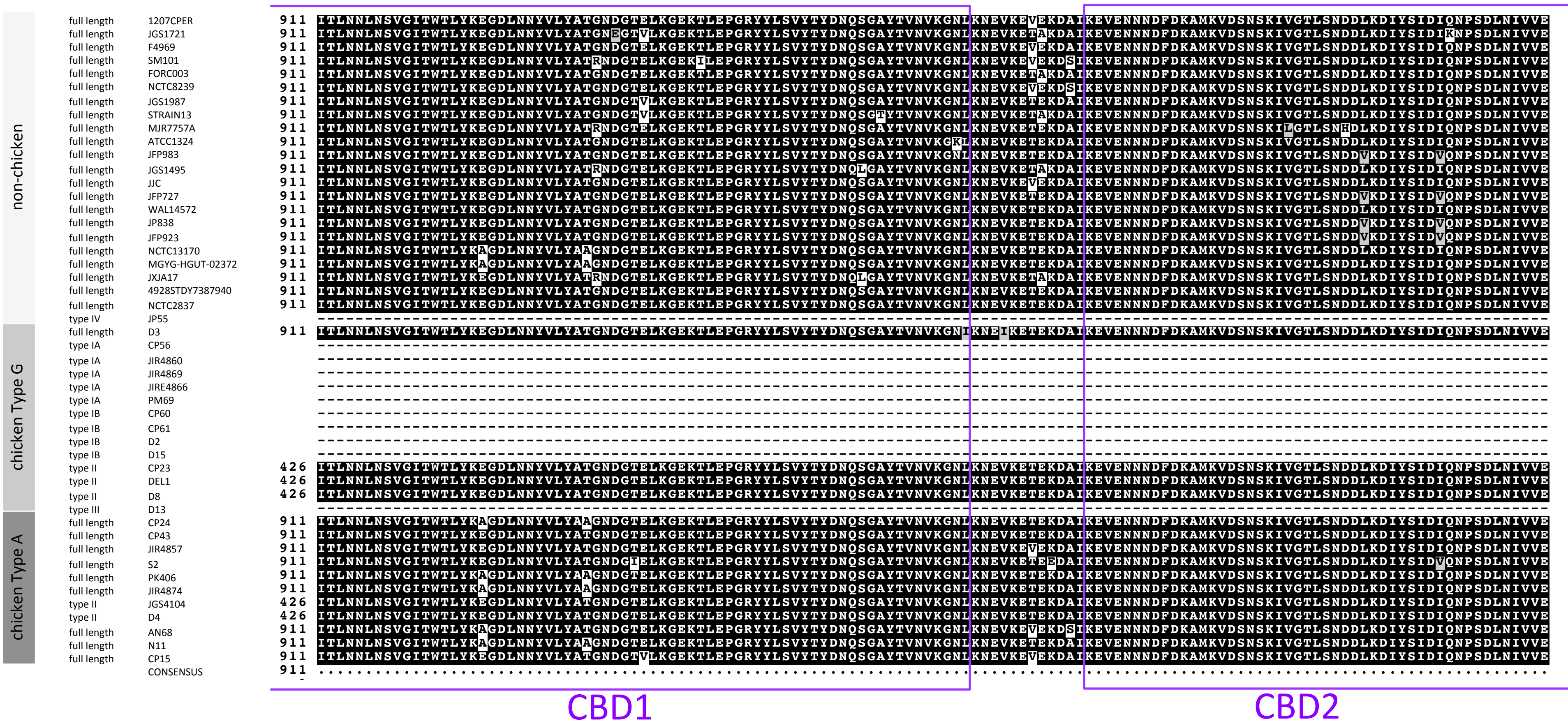

Figure S1 (cont.)

Group	Type	Strain	Position	Sequence
non-chicken	full length	1207CPER	1041	NLDNIKMNWLLYSADDLSNYVDYANADGNKLSNTCKLNPGKYYLCVYQFENSGTGNYTVNLQNK
non-chicken	full length	JGS1721	1041	NLDNIKMNWLLYSADDLSNYVDYANADGNKLSNTCKLNPGKYYLCVYQFENSGTGNYTVNLQSK
non-chicken	full length	F4969	1041	NLDNIKMNWLLYSADDLSNYVDYANADGNKLSNTCKLNPGKYYLCVYQFENSGTGNYTVNLQNK
non-chicken	full length	SM101	1041	NLDNIKMNWLLYSADDLSNYVYYANADGNKLSNTCKLNPGKYYLCVYQFENSGTGNYTVNLQNK
non-chicken	full length	FORC003	1041	NLDNIKMNWLLYSADDLSNYVDYANADGNKLSNTCKLNPGKYYLCVYQFENSGTGNYTVNLQNK
non-chicken	full length	NCTC8239	1041	NLDNIKMNWLLYSADDLSNYVDYANADGNKLSNTCKLNPGKYYLCVYQFENSGTGNYTVNLQNK
non-chicken	full length	JGS1987	1041	NLDNIKMNWLLYSADDLSNYVDYANADGNKLSNTCKLNPGKYYLCVYQFENSGTGNYTVNLQNK
non-chicken	full length	STRAIN13	1041	NLDNIKMNWLLYSADDLSNYVDYANADGNKLSNTCKLNPGKYYLCVYQFENSGTGNYIVNLQNK
non-chicken	full length	MJR7757A	1041	NSDNIKMNWLLYSADDLSNYVDYANEDGNKLSNTCKLNPGKYYLCVYQFENSGTGNYTVNLQNK
non-chicken	full length	ATCC1324	1041	NLDNIKMNWLLYSADDLSNYVDYANADGNKLSNTCKLNPGKYYLCVYQFENSGTGNYTINLQNK
non-chicken	full length	JFP983	1041	NLDNIKMNWLLYSADDLSNYVDYANTDGNKLSNTCKLNPGKYYLCVYQFENSGTGNYTVNLQSK
non-chicken	full length	JGS1495	1041	NLDNMKMNWLLYSADDLSKYVDYANADGNKLSNTCKLNPGKYYLCVYQFENSGTGNYTVNLQNK
non-chicken	full length	JJC	1041	NLDNIKMNWLLYSADDLSNYVDYANADGNKLSNTCKLNPGKYYLCVYQFENSGTGNYTVNLQNK
non-chicken	full length	JFP727	1041	NLDNIKMNWLLYSADDLSNYVDYANTDGNKLSNTCKLNPGKYYLCVYQFENSGTGNYTVNLQSK
non-chicken	full length	WAL14572	1041	NLDNIKMNWLLYSADDLSNYVDYANADGNKLSNTCKLNPGKYYLCVYQFENSGTGNYTVNLQNK
non-chicken	full length	JP838	1041	NLDNIKMNWLLYSADDLSNYVDYANTDGNKLSNTCKLNPGKYYLCVYQFENSGTGNYTVNLQSK
non-chicken	full length	JFP923	1041	NLDNIKMNWLLYSADDLSNYVDYANTDGNKLSNTCKLNPGKYYLCVYQFENSGTGNYTVNLQSK
non-chicken	full length	NCTC13170	1041	NLDNIKMNWLLYSADDLSNYVDYANADGNKLSNTCKLNPGKYYLCVYQFENSGTGNYTVNLQNK
non-chicken	full length	MGYG-HGUT-02372	1041	NLDNIKMNWLLYSADDLSNYVDYANADGNKLSNTCKLNPGKYYLCVYQFENSGTGNYTVNLQNK
non-chicken	full length	JXJA17	1041	NLDNMKMNWLLYSADDLSKYVDYANADGNKLSNTCKLNPGKYYLCVYQFENSGTGNYTVNLQNK
non-chicken	full length	4928STDY7387940	1041	NLDNIKMNWLLYSADDLSNYVDYANADGNKLSNTCKLNPGKYYLCVYQFENSGTGNYTVNLQNK
non-chicken	full length	NCTC2837	1041	NLDNIKMNWLLYSADDLSNYVDYANADGNKLSNTCKLNPGKYYLCVYQFENSGTGNYTVNLQNK
chicken Type G	type IV	JP55		--
chicken Type G	full length	D3	1041	NLDNIKMNWLLYSADDLSNYVDYANADGNKLSNTCKLNPGKYYLCVYQFENSGTGNYTVNLQNK
chicken Type G	type IA	CP56		--
chicken Type G	type IA	JIR4860		--
chicken Type G	type IA	JIR4869		--
chicken Type G	type IA	JIRE4866		--
chicken Type G	type IA	PM69		--
chicken Type G	type IB	CP60		--
chicken Type G	type IB	CP61		--
chicken Type G	type IB	D2		--
chicken Type G	type IB	D15		--
chicken Type G	type II	CP23	556	NLDNIKMNWLLYSADDLSNYVDYANADGNKLSNTCKLNPGKYYLCVYQFENSGTGNYTVNLQNK
chicken Type G	type II	DEL1	556	NLDNIKMNWLLYSADDLSNYVDYANADGNKLSNTCKLNPGKYYLCVYQFENSGTGNYTVNLQNK
chicken Type G	type II	D8	556	NLDNIKMNWLLYSADDLSNYVDYANADGNKLSNTCKLNPGKYYLCVYQFENSGTGNYTVNLQNK
chicken Type G	type III	D13		--
chicken Type A	full length	CP24	1041	NLDNIKMNWLLYSADDLSNYVDYANADGNKLSNTCKLNPGKYYLCVYQFENSGTGNYTVNLQNK
chicken Type A	full length	CP43	1041	NLDNIKMNWLLYSADDLSNYVDYANADGNKLSNTCKLNPGKYYLCVYQFENSGTGNYTVNLQNK
chicken Type A	full length	JIR4857	1041	NLDNIKMNWLLYSADDLSNYVDYANADGNKLSNTCKLNPGKYYLCVYQFENSGTGNYTVNLQNK
chicken Type A	full length	S2	1041	NLDNIKMNWLLYSADDLSNYVDYANTDGNKLSNTCKLNPGKYYLCVYQFENSGTGNYTVNLQSK
chicken Type A	full length	PK406	1041	NLDNIKMNWLLYSADDLSNYVDYANADGNKLSNTCKLNPGKYYLCVYQFENSGTGNYTVNLQNK
chicken Type A	full length	JIR4874	1041	NLDNIKMNWLLYSADDLSNYVDYANADGNKLSNTCKLNPGKYYLCVYQFENSGTGNYTVNLQNK
chicken Type A	type II	JGS4104	556	NLDNIKMNWLLYSADDLSNYVDYANADGNKLSNTCKLNPGKYYLCVYQFENSGTGNYTVNLQNK
chicken Type A	type II	D4	556	NLDNIKMNWLLYSADDLSNYVDYANADGNKLSNTCKLNPGKYYLCVYQFENSGTGNYTVNLQNK
chicken Type A	full length	AN68	1041	NSDNIKMNWLLYSADDLSNYVDYANADGNKLSNTCKLNPGKYYLCVYQFENSGTGNYTVNLQNK
chicken Type A	full length	N11	1041	NLDNIKMNWLLYSADDLSNYVDYANADGNKLSNTCKLNPGKYYLCVYQFENSGTGNYTVNLQNK
chicken Type A	full length	CP15	1041	NLDNIKMNWLLYSADDLSNYVDYANADGNKLSNTCKLNPGKYYLCVYQFENSGTGNYTVNLQNK
		CONSENSUS	1041	....

CBD2
